# Association between antenatal care facility readiness and provision of care at the client level and facility level in five low- and middle-income countries

**DOI:** 10.1186/s12913-023-10106-5

**Published:** 2023-10-17

**Authors:** Ashley Sheffel, Emily Carter, Scott Zeger, Melinda K. Munos

**Affiliations:** 1 Department of International Health, Johns Hopkins University Bloomberg School of Public Health, 615 N Wolfe St, Baltimore, MD 21205-2103 USA; 2 Departments of Biostatistics and International Health, Johns Hopkins University Bloomberg School of Public Health, 615 N Wolfe St, Baltimore, MD 21205-2103 USA

**Keywords:** Quality of care, Service readiness, Health services research, Health systems research, Maternal health, Antenatal care, International health, Developing countries, Quantitative methods

## Abstract

**Background:**

Despite growing interest in monitoring improvements in quality of care, data on service quality in low-income and middle-income countries (LMICs) is limited. While health systems researchers have hypothesized the relationship between facility readiness and provision of care, there have been few attempts to quantify this relationship in LMICs. This study assesses the association between facility readiness and provision of care for antenatal care at the client level and facility level.

**Methods:**

To assess the association between provision of care and various facility readiness indices for antenatal care, we used multilevel, multivariable random-effects linear regression models. We tested an inflection point on readiness scores by fitting linear spline models. To compare the coefficients between models, we used a bootstrapping approach and calculated the mean difference between all pairwise comparisons. Analyses were conducted at client and facility levels.

**Results:**

Our results showed a small, but significant association between facility readiness and provision of care across countries and most index constructions. The association was most evident in the client-level analyses that had a larger sample size and were adjusted for factors at the facility, health worker, and individual levels. In addition, spline models at a facility readiness score of 50 better fit the data, indicating a plausible threshold effect.

**Conclusions:**

The results of this study suggest that facility readiness is not a proxy for provision of care, but that there is an important association between facility readiness and provision of care. Data on facility readiness is necessary for understanding the foundations of health systems particularly in countries with the lowest levels of service quality. However, a comprehensive view of quality of care should include both facility readiness and provision of care measures.

**Supplementary Information:**

The online version contains supplementary material available at 10.1186/s12913-023-10106-5.

## Background

As the Sustainable Development Goals (SDGs) recognize the importance of both health-care coverage and the quality of health services [1, 2], there has been a global effort to emphasize the critical role of delivering high quality health services [3, 4]. Low-income and middle-income countries (LMICs) are tasked with monitoring progress toward improved coverage and improved service quality. However, measuring service quality is complicated by the lack of a consistent definition of quality of care and the lack of well-defined indicators for measuring quality of care for health services. As quality of care is complex, countries remain unclear about what indicators are most important for them to measure and monitor and what data sources should be utilized to do so. Global priorities are focused on reducing maternal and newborn morbidity and mortality, making the measurement of the quality of health services for pregnant women especially critical. For example, high quality antenatal care (ANC) services have the potential to reduce maternal morbidity and improve newborn survival since these services ensure women maintain a healthy pregnancy and also promote safe delivery and postnatal attendance [5–7].

Many quality of care frameworks have been proposed, most building from the seminal quality of care framework introduced by Donabedian in 1988 which characterizes quality at three levels: (1) structure — commonly called facility readiness, (the setting in which care occurs including material resources, human resources, and organizational structure), (2) process — commonly referred to as provision of care, (the quality of medical advice delivered by providers to clients, as well as interpersonal relationships between the provider and the client), and (3) outcome — the effects of care on the health status of patients, including both changes in health status and health behavior, as well as improvements in patient knowledge and the degree of the patient’s satisfaction with care [8]. Donabedian theorized that there is a relationship between facility readiness, provision of care, and health outcomes; high facility readiness increases the likelihood of good provision of care, and good provision of care increases the likelihood of a good outcome [8, 9]. Having the essential facility readiness components is necessary to provide a quality health care service, but not sufficient to guarantee quality care. It is the provision of care, or the set of activities, that transforms facility inputs into improved health outcomes for patients.

Despite the increasing focus on quality of care, data on service quality in LMICs is limited [10, 11]. Increasingly, health facility assessments (HFAs) are being conducted in LMICs to try to fill this data gap [12]. HFAs are a rich source of information about the performance of health systems yet are variable in terms of the information collected on quality of care. Two of the most widely implemented HFAs are the Service Provision Assessment (SPA) and the Service Availability and Readiness Assessment (SARA) [13, 14]. SPA and SARA surveys both capture data on facility readiness across a wide range of health services through implementation of a facility audit. However, only the SPA collects data on provision of care for a limited set of health services through implementation of direct observation of client visits and patient exit interviews [13, 14]. In addition to the SPA, there are several service-specific surveys implemented in LMICs that collect provision of care data such as the Quick Investigation of Quality (QIQ) tool, which assesses family planning, and the Needs Assessments for Emergency Obstetric and Newborn Care (EmONC), which assess delivery and newborn care [15, 16]. The more limited availability of provision of care data can be attributed to the challenges in collecting this data as it is both resource-intensive and logistically challenging to implement [17, 18]. There has been interest in using facility readiness as a potential proxy for provision of care since it is easier to collect. A recent study found that facility-level correlation between readiness and provision of care is low [19]. As a result, the recent The Lancet Global Health Commission on High Quality Health Systems in the SDG Era recommended focusing on provision of care measures when assessing quality of care [3].

While health systems researchers have hypothesized the relationship between facility readiness and provision of care, there have been few attempts to quantify this relationship in LMICs. A number of studies have attempted to identify determinants of service quality in LMICs. Studies have identified associations between service quality and service volume [20, 21], and between various domains of quality [22], but not between structural and process quality. Understanding this association is important for both measurement of quality of care and quality improvement processes. This study builds on the work by Leslie et al. (2017) [19] and incorporates a client-level analysis which allows for adjustment for facility and client level characteristics as well as the use of multiple approaches to generate readiness indices. This study aims to assess the association between facility readiness and provision of care for ANC at the client level and facility level utilizing multiple methods for generating facility readiness indices and adjusting for facility-level and client-level characteristics.

## Methods

### Data

#### Service provision assessment (SPA)

This analysis used data from the SPA to generate nationally representative data on health service delivery [13, 23]. The SPA includes a standard set of survey instruments: a facility inventory questionnaire, health worker interviews, observation of ANC consultations, and exit interviews with ANC clients.

We examined all SPA surveys for inclusion in the analysis (total of 31). We included all SPA surveys that were available for public use as of early 2018 which used the Demographic and Health Surveys (DHS)-VI or DHS-VII questionnaire (14 surveys excluded), that included observations of ANC consultations (six surveys excluded), were conducted in the last 10 years (between 2012 and 2022; two surveys excluded), and were the most recent survey for the country meeting inclusion criteria (1 survey excluded). The included surveys are from Haiti (2013), Malawi (2013/2014), Nepal (2015), Senegal (2016), and Tanzania (2014/2015). Comprehensive information on the survey methodology and questionnaires is detailed in the SPA final country reports [24–28]. In Nepal, Senegal, and Tanzania, the survey was a nationally representative sample of health facilities selected using stratified systematic probability sampling with stratification by administrative area (geo-ecological region and development-ecological zone in Nepal, and region in Tanzania) and facility type (with oversampling of some facility types such as hospitals). In Haiti and Malawi, the survey was comprised of a national census of all health facilities. The facility inventory module was completed by all surveyed facilities in the five countries. Additionally, up to eight health workers were interviewed within each facility. Selected health workers include those whose consultations were observed and those who provided information for any section of the inventory questionnaire. Sampling of clients for observation was done using systematic sampling and was dependent on the number of clients present at each service site on the day of the visit. For facilities where the number of ANC clients could not be anticipated, opportunistic sampling was used when clients arrived. At a minimum, five client observations were completed per service provider, with a maximum of 15 observations in any given facility for each service. Client exit interviews were conducted following each client-provider observation.

#### Analysis

In order to standardize expected clinical actions, we limited this analysis to facilities offering ANC services with at least one first ANC client observation, and to women attending the health facility for a first ANC visit. Of the total number of facilities offering ANC services, the percentage which also included any ANC client observations ranged from 52% in Nepal to 90% in Senegal. After additionally restricting to facilities with first visit ANC client observations and complete cases, the percentage of facilities offering ANC included in the analysis ranged from 32% in Nepal to 55% in Senegal. We did not include observations containing incomplete data. Supplementary Tables 1, Additional File 1 provides information on the full sample size and analytical sample size for each country.

To assess the effect of excluding incomplete cases, we compared facilities, health workers, and ANC clients with and without complete data across background characteristics. For continuous variables, we calculated means and used t-tests to assess differences between groups. For categorical variables, we calculated proportions and used chi-square tests to assess differences between groups. There were no statistically significant differences between the groups. Supplementary Tables 2, Additional File 1 provides the details of the all cases versus complete cases analysis.

#### Facility readiness and provision of care indices

**Fig. 1:**
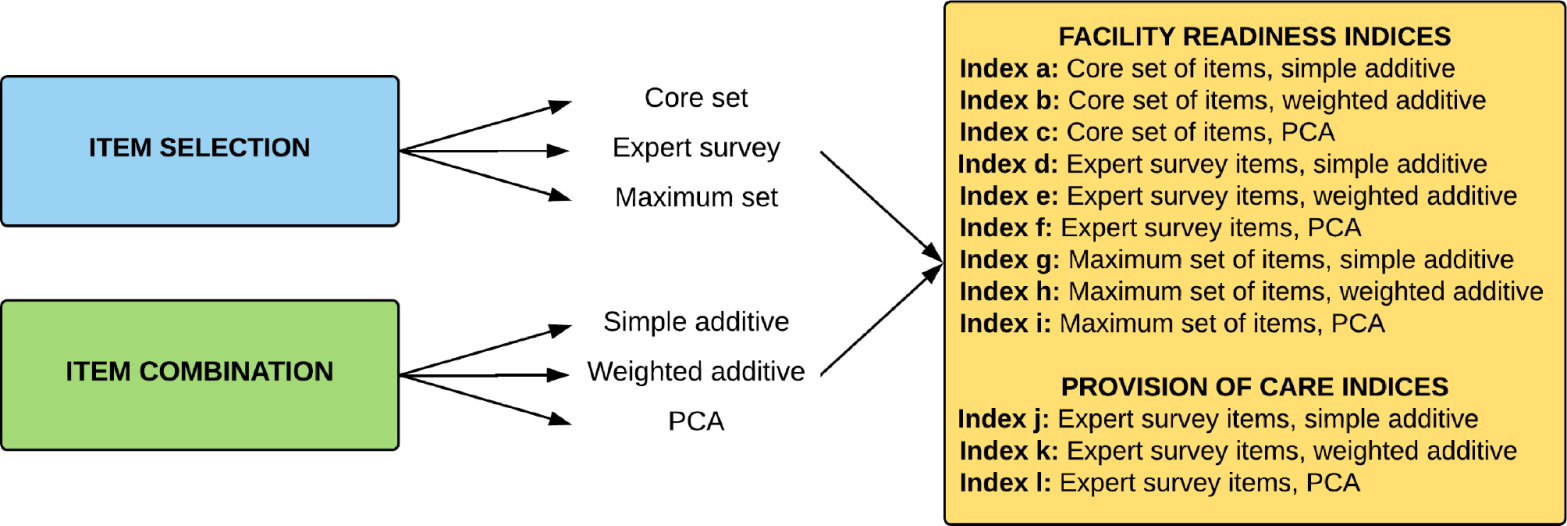
Process of Index Creation

As described previously by Sheffel et al., we created nine indices for facility readiness using three methods for selecting items (core set of items, expert survey set of items, and maximum set of items) and three methods for combining items (simple additive, weighted additive, and principal component analysis (PCA)) [29]. These methods are commonly utilized across service areas to create summary indices of service quality, yet there is no consensus on a single best approach [12, 29–31]. In addition, we created a provision of care index using the expert survey set of items for selecting items and a weighted approach to combine items (Fig. 1). A detailed description of item selection, item combination, and index creation are described in Sheffel et al. [29]. Briefly described, the core set of items (21 readiness items) was identified by reviewing the provision of care items required for an ANC visit based on WHO FANC guidelines and WHO recommendations on ANC for a positive pregnancy experience, and by determining the human resources, equipment and supplies, medicines, and diagnostics required to deliver each specific item. The expert set of items (19 readiness items, 49 provision of care items) was identified using results from an expert survey whereby items rated by the expert group as essential were included. The maximum set of items (38 readiness items) included all items identified in the SPA related to ANC readiness across the following domains: human resources, equipment and supplies, medicines, diagnostics, and basic amenities. For each index, the possible range of scores was from 0 to 100%. The distribution of readiness scores and provision of care scores for each country are presented in Supplementary Fig. 1, Additional File 1 and Supplementary Fig. 2, Additional File 1.

#### Standardization of facility and health worker level variables

Facility-level variables such as facility type and managing authority were standardized across the five surveys. For facility type, three categories were constructed — hospital, health center/clinic, and dispensary. Since facility size is often associated with facility type, we conducted descriptive analyses of the number of total staff and the number of inpatient beds per facility type in order to determine which facility types were most similar in size. Supplementary Tables 3, Additional File 1 provides details of this analysis. For managing authority, we created three categories — government/public, private (non-faith based), and private faith based. At the health worker level, qualification was standardized across the surveys into three categories — physicians, clinical officers, and nurses/midwives (Supplementary Text, Additional File 1 provides cadre definitions).

#### Additional covariates

We included facility, health worker, and individual characteristics as covariates in our regression models. Facility-level covariates included facility type, managing authority, urbanicity, and average number of staff. Health worker-level covariates included qualification and gender. Individual-level covariates included the number of weeks pregnant, if the client had a previous pregnancy, age, and the highest level of education attained by the client. For Nepal, the covariate urbanicity was excluded as this data was not collected in the Nepal survey. For Haiti and Senegal, the covariate number of weeks pregnant was excluded because more than 50% of the data was missing for this item.

#### Regression analysis

To assess the association between provision of care and facility readiness, we first conducted a bivariate regression of provision of care on each facility readiness index described above. Next, we used a multilevel, multivariable linear regression model with random effects for facility and health worker, controlling for facility, health worker, and individual characteristics. We ran nine separate models, one for each facility readiness index. The outcome in each case was the same provision of care index. Analyses were conducted separately for each country. Visual examination of the data suggested a possible inflection point around a readiness score of 50 (see Supplementary Figs. 3–12, Additional File 1); we tested this by fitting linear spline models with a single knot at 50 for all countries except for Senegal where there were few facilities with readiness scores below 50. For each model, we divided the coefficient for the readiness index by the standard error to obtain a measure of the strength of the association that accounts for both the estimate and the standard error.

We compare the readiness coefficients obtained from the client-level analyses to quantify the difference in the strength of association between readiness and provision of care using the different approaches for item selection and item aggregation. We use bootstrapping to non-parametrically estimate the joint distribution of these two coefficients and to estimate their mean difference and its standard error [32, 33]. For each dataset, we generated 500 independent survey samples by resampling facilities with replacement, selecting the same number of facilities as in the original survey. Then, we merged the sampled facilities with client data to get sampled clients for the client-level analysis. For each of the 500 samples, we ran the nine linear models and nine linear spline models. We then calculated the mean difference of the coefficient for facility readiness and the standard error of the mean difference for each of the 36 pairwise comparisons. Statistical significance was determined using a Bonferroni adjusted p-value to account for the multiple pairwise comparisons.

Finally, we collapsed the client-level dataset into a facility-level dataset by taking the mean provision of care index score across clients within a facility and conducted a multivariable linear regression adjusting for facility characteristics. We compare the readiness coefficient obtained from the common facility-level regression with its analogue from the client-level analysis to quantify the bias resulting from failure to control for client-level confounders and other model misspecifications. We use facility-level bootstrapping to non-parametrically estimate the joint distribution of these two coefficients and to estimate their mean difference and its standard error [32, 33].

All statistical analyses were carried out using R version 4.1.3 [34]. We did not include survey weights to account for the complex survey design in the regression analyses. There is considerable debate among survey experts regarding the best approach for regression analysis with complex survey data [35–38]. Given that this paper aimed to explore the client-level relationship between readiness and provision of care and not to estimate a population mean, we followed the common practice of opting not to weight the data by the complex survey design weights to avoid unnecessarily increasing the variance of regression coefficients and thereby decreasing the study power. By virtue of not using weights, primary level facilities that are underrepresented in the sample are not upweighted in the regression analyses. However, adjusting for facility type and managing authority in the regression models mitigates this potential bias.

## Results

### Background characteristics

The final analytic sample consisted of 358 health facilities, 406 health workers and 779 ANC clients in Haiti; 253 health facilities, 283 health workers, and 815 ANC clients in Malawi; 282 health facilities, 337 health workers, and 520 ANC clients in Nepal; 179 health facilities, 184 health workers, and 297 ANC clients in Senegal; and 632 health facilities, 799 health workers, and 1,681 ANC clients in Tanzania (Supplementary Table 1, Additional File 1). In all countries, most health facilities were government-run health centers, and in all countries except Nepal the majority of health facilities were rural. In addition, health workers were predominantly female nurses and midwives, with the minority of providers being physicians and clinical officers. More than half of ANC clients were primigravida clients, and the majority had at least a primary education except for in Senegal where just under half of clients had at least a primary education (Table 1).

**Table 1:** Background Characteristics (Haiti, Malawi, Nepal, Senegal, and Tanzania)

	Haiti	Malawi	Nepal	Senegal	Tanzania
**Facility characteristics**	**N = 358**	** N = 253**	** N = 282**	** N = 179**	** N = 632**
Facility type					
Hospital	19.7%	24.8%	42.5%	9.7%	26.8%
Health center/clinic	51.3%	72.8%	33.5%	18.3%	39.1%
Dispensary	29.0%	2.4%	24.0%	72.0%	34.1%
Managing authority					
Government	45.1%	71.5%	83.6%	91.4%	73.5%
Private non-faith based	36.1%	6.5%	15.6%	8.6%	6.8%
Private faith based	18.9%	22.0%	0.7%		19.6%
Urban/rural					
Urban	45.1%	20.3%		46.3%	32.3%
Rural	54.9%	79.7%		53.7%	67.7%
Average number of staff	12.6	7.8	10.5	7.7	10.4
**Health worker characteristics**	**N = 406**	** N = 283**	** N = 337**	** N = 184**	** N = 799**
Qualification					
Physician	42.4%	0.0%	16.7%	1.1%	1.8%
Clinical officer	0.0%	5.1%	5.5%	11.7%	3.2%
Nurse/Midwife	57.6%	94.9%	77.9%	87.2%	95.0%
Gender					
Male	32.5%	24.7%	11.2%	10.0%	12.4%
Female	67.5%	75.3%	88.8%	90.0%	87.6%
**Individual characteristics**	**N = 779**	** N = 815**	** N = 520**	** N = 297**	** N = 1681**
Number of weeks pregnant		21.3	19.7		22.4
Client had a previous pregnancy	33.4%	24.9%	48.5%	24.2%	25.7%
Age (years)	26.6	25.1	22.9	25.8	25.9
Highest level of education attained					
Never attended school	14.1%	13.7%	25.0%	52.5%	18.5%
Primary	39.9%	64.2%	8.3%	25.6%	60.1%
Secondary	43.6%	20.0%	48.1%	19.9%	19.1%
Higher	2.3%	2.1%	18.7%	2.0%	2.3%

### Association between facility readiness and provision of care at the client level

The bivariate analysis results are presented in Supplementary Table 4, Supplementary Table 5, and Supplementary Figs. 3–12; Additional File 1. The adjusted association between facility readiness and provision of care at the client level is shown in Table 2. In Haiti, Malawi, and Tanzania the majority of readiness indices were statistically significantly associated with provision of care while in Nepal and Senegal three out of nine readiness indices were statistically significantly associated with provision of care. However, across all countries the effect sizes were relatively small. The effect size for the measure of association between facility readiness and provision of care at the client level across all countries ranged from 0.05 (core PCA, Nepal) to 0.39 (maximum PCA, Senegal). This range of effect sizes indicates that for every 10-point increase in readiness, provision of care increased by 0.5 points (core PCA, Nepal) to 3.9 points (maximum PCA, Senegal) on a 100-point scale. There was no discernable pattern in terms of which types of readiness indices were more strongly associated with provision of care.

**Table 2:** Multilevel, Random Effects Model of the Association between Readiness and Provision of Care, Haiti, Malawi, Nepal, Senegal, and Tanzania

**Haiti**				
	Estimate	Std. Error	p-value	Estimate / Std. Error
Core simple	0.136	0.038	0.000*	3.534
Core weighted	0.078	0.032	0.016*	2.419
Core PCA	0.073	0.029	0.011*	2.549
Expert simple	0.111	0.036	0.002*	3.057
Expert weighted	0.105	0.033	0.002*	3.133
Expert PCA	0.080	0.030	0.007*	2.715
Maximum simple	0.151	0.043	0.001*	3.485
Maximum weighted	0.124	0.039	0.002*	3.161
Maximum PCA	0.099	0.040	0.013*	2.484
**Malawi**				
	Estimate	Std. Error	p-value	Estimate / Std. Error
Core simple	0.165	0.060	0.006*	2.750
Core weighted	0.063	0.052	0.222	1.223
Core PCA	0.135	0.052	0.011*	2.570
Expert simple	0.210	0.062	0.001*	3.384
Expert weighted	0.173	0.056	0.002*	3.110
Expert PCA	0.205	0.062	0.001*	3.284
Maximum simple	0.191	0.066	0.004*	2.893
Maximum weighted	0.224	0.070	0.002*	3.194
Maximum PCA	0.199	0.066	0.003*	2.987
**Nepal**				
	Estimate	Std. Error	p-value	Estimate / Std. Error
Core simple	0.103	0.054	0.060	1.892
Core weighted	0.085	0.050	0.088	1.712
Core PCA	0.049	0.044	0.271	1.103
Expert simple	0.102	0.058	0.077	1.773
Expert weighted	0.067	0.058	0.244	1.168
Expert PCA	0.054	0.049	0.279	1.084
Maximum simple	0.150	0.067	0.026*	2.236
Maximum weighted	0.131	0.062	0.034*	2.126
Maximum PCA	0.136	0.059	0.022*	2.305
**Senegal**				
	Estimate	Std. Error	p-value	Estimate / Std. Error
Core simple	0.172	0.078	0.029*	2.197
Core weighted	0.115	0.053	0.030*	2.183
Core PCA	0.189	0.116	0.105	1.627
Expert simple	0.136	0.083	0.102	1.642
Expert weighted	0.050	0.061	0.416	0.815
Expert PCA	0.129	0.195	0.510	0.659
Maximum simple	0.285	0.095	0.003*	3.015
Maximum weighted	0.136	0.084	0.107	1.622
Maximum PCA	0.390	0.210	0.065	1.857
**Tanzania**				
	Estimate	Std. Error	p-value	Estimate / Std. Error
Core simple	0.206	0.034	0.000*	5.998
Core weighted	0.131	0.029	0.000*	4.601
Core PCA	0.172	0.031	0.000*	5.491
Expert simple	0.163	0.034	0.000*	4.784
Expert weighted	0.174	0.034	0.000*	5.156
Expert PCA	0.115	0.029	0.000*	4.013
Maximum simple	0.175	0.040	0.000*	4.358
Maximum weighted	0.198	0.038	0.000*	5.199
Maximum PCA	0.169	0.039	0.000*	4.348

**Note:** Model is a linear mixed effects model with random intercepts for facility and health worker, controlling for individual, health worker, and facility characteristics; client-level analysis

* p < 0.05

The comparison of each of the client-level index estimates of association is shown in Supplementary Table 6, Additional File 1. There were few significant differences between the coefficients from different models, indicating that the approaches to item selection and item combination utilized for readiness index construction ultimately had little impact on the relationship between readiness and provision of care. Across all countries, there was a total of 19-pairwise comparisons with statistically significant differences. Of the 36-pairwise comparisons in each country, Tanzania had the most statistically significant differences (9) while Nepal had the fewest statistically significant differences (1). The index comparisons that were significantly different most often used different approaches for combining the same set of items (9/19 significantly different comparisons).

**Fig. 2:**
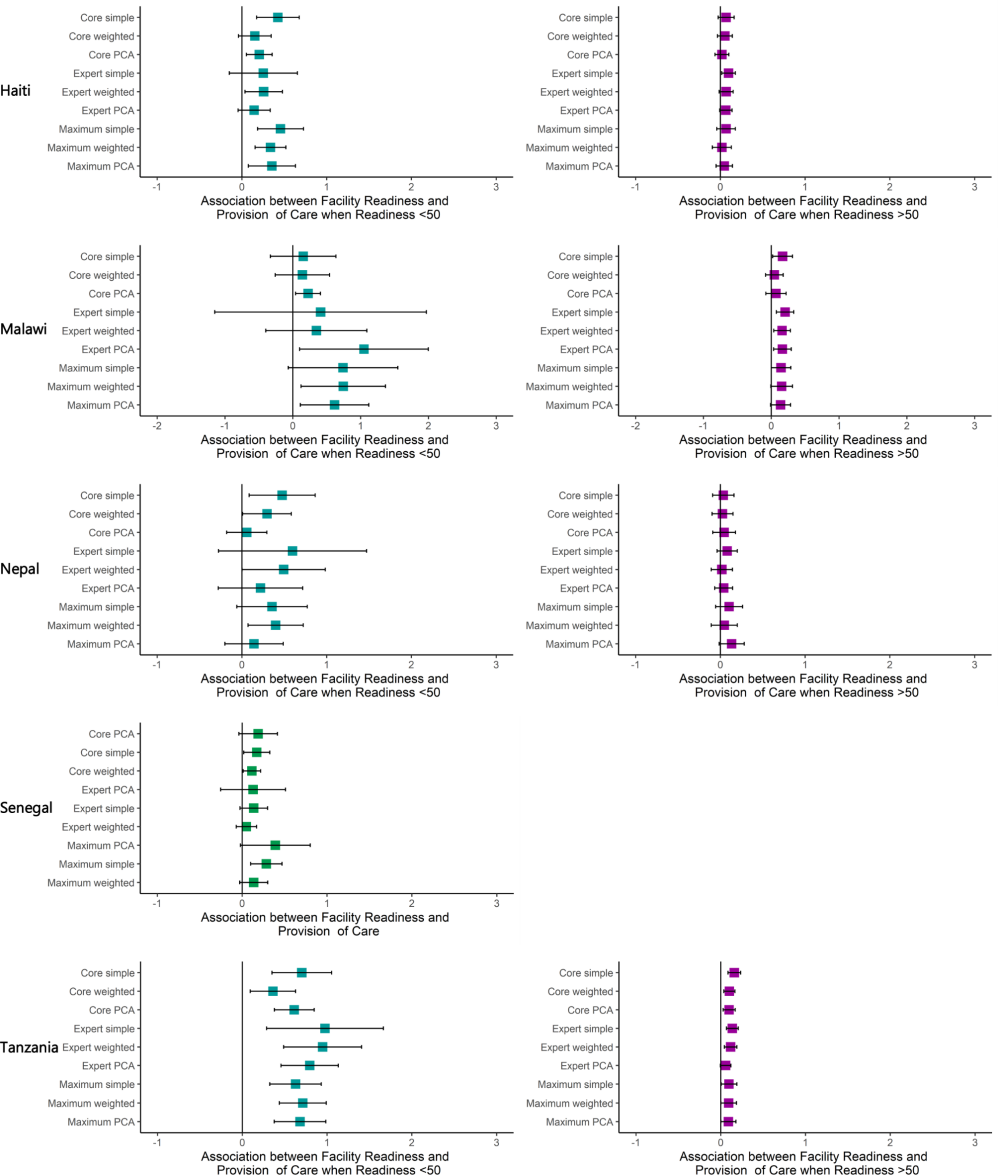
Multilevel, Random Effects Model: Association between Readiness and Provision of Care with Spline at 50

The adjusted associations between facility readiness and provision of care with a linear spline at 50 at the client level are shown in Table 3; Fig. 2. Adding the spline resulted in larger coefficients, relative to the models without a spline, when the facility readiness score was less than 50, and in smaller coefficients when the readiness score was greater than 50 for almost all models. For example, in Tanzania, when the facility readiness score was less than 50, the measure of association between facility readiness and provision of care at the client level ranged from 0.362 (core weighted) to 0.976 (expert simple) and was statistically significant for all nine indices. When the facility readiness score was more than 50, the measure of association between facility readiness and provision of care at the client level ranged from 0.058 (expert PCA) to 0.16 (core simple) and was statistically significant for eight out of the nine indices.

**Table 3:** Multilevel, Random Effects Model of the Association between Readiness and Provision of Care with a Spline at 50, Haiti, Malawi, Nepal, and Tanzania

**Haiti**										
	N Ready < = 50	Estimate < 50	Std. Error	p-value	Estimate /Std. Error	N Ready > 50	Estimate > 50	Std. Error	p-value	Estimate /Std. Error
Core simple	151	0.425	0.128	0.001*	3.310	628	0.068	0.048	0.155	1.425
Core weighted	138	0.152	0.098	0.123	1.545	641	0.053	0.044	0.231	1.201
Core PCA	298	0.205	0.078	0.009*	2.634	481	0.018	0.042	0.662	0.437
Expert simple	76	0.253	0.205	0.218	1.233	703	0.097	0.041	0.018*	2.372
Expert weighted	139	0.256	0.113	0.023*	2.275	640	0.068	0.042	0.105	1.624
Expert PCA	186	0.145	0.097	0.136	1.492	593	0.065	0.037	0.083	1.739
Maximum simple	150	0.456	0.138	0.001*	3.305	629	0.068	0.056	0.225	1.215
Maximum weighted	233	0.338	0.093	0.000*	3.649	546	0.018	0.057	0.751	0.317
Maximum PCA	152	0.354	0.142	0.013*	2.489	627	0.046	0.049	0.353	0.930
**Malawi**										
	N Ready < = 50	Estimate < 50	Std. Error	p-value	Estimate /Std. Error	N Ready > 50	Estimate > 50	Std. Error	p-value	Estimate /Std. Error
Core simple	180	0.154	0.247	0.533	0.624	635	0.168	0.075	0.026*	2.238
Core weighted	102	0.143	0.205	0.486	0.698	713	0.047	0.066	0.480	0.707
Core PCA	522	0.225	0.093	0.016*	2.420	293	0.070	0.076	0.358	0.921
Expert simple	15	0.411	0.796	0.606	0.516	800	0.205	0.066	0.002*	3.118
Expert weighted	70	0.348	0.380	0.361	0.915	745	0.160	0.063	0.011*	2.548
Expert PCA	70	1.049	0.484	0.031*	2.168	745	0.165	0.066	0.013*	2.507
Maximum simple	112	0.741	0.412	0.074	1.797	703	0.145	0.074	0.051	1.965
Maximum weighted	119	0.745	0.317	0.020*	2.346	696	0.154	0.081	0.059	1.897
Maximum PCA	170	0.616	0.258	0.018*	2.391	645	0.138	0.075	0.069	1.827
**Nepal**										
	N Ready < = 50	Estimate < 50	Std. Error	p-value	Estimate /Std. Error	N Ready > 50	Estimate > 50	Std. Error	p-value	Estimate /Std. Error
Core simple	81	0.475	0.198	0.017*	2.394	439	0.034	0.064	0.594	0.534
Core weighted	68	0.296	0.146	0.044*	2.022	452	0.025	0.063	0.690	0.400
Core PCA	115	0.058	0.121	0.635	0.476	405	0.044	0.069	0.519	0.645
Expert simple	15	0.598	0.446	0.182	1.340	505	0.080	0.061	0.190	1.314
Expert weighted	39	0.492	0.250	0.050	1.967	481	0.018	0.064	0.785	0.274
Expert PCA	37	0.219	0.254	0.390	0.862	483	0.040	0.054	0.464	0.734
Maximum simple	91	0.356	0.212	0.095	1.677	429	0.103	0.081	0.204	1.274
Maximum weighted	115	0.398	0.166	0.017*	2.392	405	0.046	0.079	0.561	0.582
Maximum PCA	86	0.143	0.176	0.415	0.816	434	0.133	0.076	0.079	1.765
**Tanzania**										
	N Ready < = 50	Estimate < 50	Std. Error	p-value	Estimate /Std. Error	N Ready > 50	Estimate > 50	Std. Error	p-value	Estimate /Std. Error
Core simple	112	0.701	0.179	0.000*	3.909	1569	0.160	0.038	0.000*	4.237
Core weighted	149	0.362	0.137	0.008*	2.646	1532	0.102	0.033	0.002*	3.046
Core PCA	198	0.613	0.120	0.000*	5.126	1483	0.099	0.036	0.007*	2.716
Expert simple	50	0.976	0.351	0.006*	2.778	1631	0.137	0.036	0.000*	3.830
Expert weighted	88	0.947	0.235	0.000*	4.038	1593	0.116	0.038	0.002*	3.073
Expert PCA	130	0.795	0.172	0.000*	4.611	1551	0.058	0.032	0.070	1.818
Maximum simple	252	0.628	0.155	0.000*	4.061	1429	0.098	0.047	0.040*	2.064
Maximum weighted	260	0.714	0.141	0.000*	5.064	1421	0.094	0.047	0.045*	2.013
Maximum PCA	222	0.681	0.155	0.000*	4.397	1459	0.091	0.045	0.045*	2.011

**Note:** Model is a linear mixed effects model with random intercepts for facility and health worker and a spline at 50, controlling for individual, health worker, and facility characteristics; client-level analysis

* p < 0.05.

The comparison of each of the client-level spline model estimates of association (using a bootstrapping approach) is shown in Supplementary Table 7, Additional File 1. There were few statistically significant differences between coefficients. Across all countries (except Senegal), there was a total of 4-pairwise comparisons with statistically significant differences, one occurring when facility readiness was less than 50 and three occurring when facility readiness was greater than 50. Of the 72-pairwise comparisons in each country, only Tanzania and Nepal had any statistically significant differences, three and one respectively. The index comparisons that were significantly different most often used different approaches for combining the same set of items (1/1 significantly different comparison when facility readiness was less than 50; 2/3 significantly different comparisons when facility readiness was greater than 50).

### Association between facility readiness and provision of care at the facility level

The adjusted association between facility readiness and provision of care at the facility level is shown in Table 4. At the facility level, the effect size for the association between facility readiness and provision of care was similar to that seen in the client-level analysis. However, fewer facility readiness indices showed a statistically significant association with provision of care. Notably, collapsing to a facility-level analysis reduced the sample size in Haiti from 779 clients to 358 facilities, in Malawi from 815 clients to 253 facilities, and in Nepal from 520 clients to 282 facilities. In Tanzania, the facility-level sample size remained fairly large (632 facilities) and the measure of association between facility readiness and provision of care at the facility level was statistically significant for all nine indices. In Senegal, the facility-level sample size remained fairly small (179 facilities, 297 clients).

**Table 4:** Facility Level Linear Model of the Association between Readiness and Provision of Care, Haiti, Malawi, Nepal, Senegal, and Tanzania

**Haiti**				
	Estimate	Std. Error	p-value	Estimate / Std. Error
Core simple	0.099	0.039	0.012*	2.526
Core weighted	0.050	0.033	0.126	1.534
Core PCA	0.042	0.030	0.155	1.426
Expert simple	0.077	0.037	0.040*	2.063
Expert weighted	0.071	0.034	0.039*	2.070
Expert PCA	0.051	0.030	0.097	1.663
Maximum simple	0.120	0.044	0.007*	2.728
Maximum weighted	0.090	0.040	0.026*	2.241
Maximum PCA	0.063	0.041	0.125	1.536
**Malawi**				
	Estimate	Std. Error	p-value	Estimate / Std. Error
Core simple	0.170	0.060	0.005*	2.819
Core weighted	0.075	0.051	0.145	1.461
Core PCA	0.134	0.053	0.011*	2.552
Expert simple	0.214	0.061	0.001*	3.482
Expert weighted	0.184	0.055	0.001*	3.349
Expert PCA	0.204	0.062	0.001*	3.281
Maximum simple	0.184	0.066	0.006*	2.798
Maximum weighted	0.221	0.069	0.002*	3.187
Maximum PCA	0.190	0.067	0.005*	2.850
**Nepal**				
	Estimate	Std. Error	p-value	Estimate / Std. Error
Core simple	0.098	0.056	0.082	1.748
Core weighted	0.088	0.052	0.093	1.683
Core PCA	0.043	0.046	0.341	0.953
Expert simple	0.095	0.060	0.112	1.596
Expert weighted	0.063	0.060	0.293	1.054
Expert PCA	0.049	0.051	0.343	0.950
Maximum simple	0.129	0.070	0.068	1.833
Maximum weighted	0.124	0.064	0.055	1.925
Maximum PCA	0.111	0.061	0.071	1.816
**Senegal**				
	Estimate	Std. Error	p-value	Estimate / Std. Error
Core simple	0.134	0.076	0.079	1.765
Core weighted	0.097	0.051	0.059	1.904
Core PCA	0.131	0.111	0.242	1.175
Expert simple	0.111	0.081	0.173	1.367
Expert weighted	0.048	0.058	0.414	0.818
Expert PCA	0.064	0.181	0.726	0.351
Maximum simple	0.231	0.093	0.014*	2.480
Maximum weighted	0.106	0.081	0.192	1.309
Maximum PCA	0.277	0.200	0.169	1.382
**Tanzania**				
	Estimate	Std. Error	p-value	Estimate / Std. Error
Core simple	0.213	0.036	0.000*	5.831
Core weighted	0.135	0.031	0.000*	4.417
Core PCA	0.178	0.033	0.000*	5.404
Expert simple	0.166	0.036	0.000*	4.662
Expert weighted	0.187	0.036	0.000*	5.219
Expert PCA	0.121	0.030	0.000*	4.050
Maximum simple	0.177	0.042	0.000*	4.193
Maximum weighted	0.208	0.041	0.000*	5.123
Maximum PCA	0.174	0.041	0.000*	4.237

Model is a linear model, controlling for facility characteristics; facility-level analysis

* p < 0.05.

The comparison of each of the facility-level index estimates of association to the client-level index estimates of association are shown in Supplementary Table 8, Additional File 1. Across all countries, the nine comparisons showed that none of the coefficients were statistically significantly different between the client-level models and the facility-level models. However, some models showed a loss of information when collapsing from a client-level to a facility-level dataset, particularly in Haiti, where the facility-level analysis was 1.1 to 1.8 times less efficient than the client-level analysis. All other countries had less information loss in the facility-level model.

The adjusted association between facility readiness and provision of care with a linear spline at 50 at the facility-level is shown in Supplementary Table 9, Additional File 1. Similar to the client-level models, adding the spline resulted in larger coefficients, relative to the models without a spline, when facility readiness was less than 50, and in smaller coefficients when readiness was greater than 50. However, fewer of the associations were statistically significant. This may be partially due to the smaller sample size in some indices, especially in the facility readiness less than 50 group. For example, in Malawi, six of the nine indices had less than 40 observations when readiness was less than 50.

The comparison of each of the facility level spline model index estimates of association to the client-level spline model index estimates of association is shown in Supplementary Table 10, Additional File 1. Across all countries, none of the estimates of association were statistically significantly different between the client-level models and the facility-level models. However, as with the non-spline models, some models showed a loss of information when collapsing from a client-level to a facility-level dataset, particularly in Haiti, where the facility-level spline analysis would require up to 1.4 times the number of observations in order to do as well as the client-level analysis. All other countries had less information loss in the facility-level spline models.

## Discussion

This study assessed the association between facility readiness and provision of care for antenatal care at the client level and facility level in five LMICs. We found a small but significant association between facility readiness and provision of care across countries and most index constructions. Across the five countries, a ten-point increase in facility readiness was associated with a one- to two-point increase in provision of care score for most indices. This is consistent with the findings by Leslie et al. (2017) which found a positive but weak correlation between readiness and provision of ANC [19]. In our analysis, this association was strongest in the client-level analyses that had a larger sample size and adjusted for factors at the facility, health worker, and individual levels. In addition, we found that the models with a spline at a facility readiness score of 50 fit the data better, suggesting a threshold effect that is conceptually plausible. Across four of the five countries (Haiti, Malawi, Nepal, and Tanzania), the client-level and facility-level analyses found that when facility readiness was less than 50, a ten-point increase in facility readiness was associated with a one- to ten-point increase in provision of care score. This finding diverges from the findings by Leslie et al. (2017) which did not identify a significant inflection point [19].

We found that the majority of the facility readiness indices were associated with provision of care and there were several facility readiness indices that were strongly associated with provision of care in all countries, including the core simple facility readiness index. These findings may be helpful for countries in selecting an approach for creating quality of care summary indices. Based on these findings, a pragmatic approach that uses simple metrics that are easily calculated, interpreted, and adapted at country level may be warranted.

We also found that the association between readiness and provision of care held at the facility level, but there was a small loss of information when analyzing provision of care data at facility level compared to the client level. However, in some cases this tradeoff may be worthwhile in order to facilitate interpretation and use of data for decision-making. For both global and national monitoring of quality improvements, it may be more meaningful to report the overall quality of a facility as opposed to the aggregate quality of patient-provider interactions across facilities. Similarly, effective coverage is being used increasingly as a metric for monitoring progress towards universal health coverage (UHC) globally [39–44]. Many efforts to estimate effective coverage have linked household survey data on coverage of interventions with facility-level data on quality of care [45–51]. This analysis contributes to the methodological evidence-base for how to measure quality of care for use in estimating effective coverage.

While recent efforts have championed a shift towards measuring provision of care, which continues to be a significant data gap, our results highlight the continued importance of facility readiness, especially in countries that are believed to have poor quality health systems. We found that there was a strong association between readiness and provision of care when facility readiness scores were below 50. This finding is consistent with conceptual frameworks for quality of care which hypothesize that facility readiness is important and to some extent explains process quality, but that process quality is also affected by many other factors including client volume, provider motivation, provider competence, and health system management practices [17, 52–58]. Our results suggest that, while facility readiness is not a proxy for provision of care, there is a minimum threshold of facility inputs required for health care workers to deliver high quality ANC services. Below the threshold of 50, increases in readiness are associated with increases in provision of care as health care workers are gaining the necessary inputs to deliver a service. However, beyond the threshold, facility inputs are no longer driving the quality of care received. It is likely that once the minimum threshold is reached, it is what the health care workers do with facility inputs that is a larger determinant of the quality of care.

There are significant challenges to collecting provision of care data in LMICs using direct observation of clinical care. Direct observation of client visits requires highly clinically trained data collectors such as doctors or nurses to observe the consultations. In addition, it requires that patients come to the health facility seeking care for the services of interest on the day of the survey. This can be difficult in small, low-volume facilities where patient volume is insufficient to meet survey sample sizes in a timely manner [59]. It is perhaps not surprising that these smaller facilities also often deliver lower quality care, and their exclusion is problematic for obtaining accurate estimates of quality of care. For example, a study by Kruk et al. found that the quality of maternal care was substantially lower in primary care facilities as compared to secondary care facilities and that low delivery volume was consistently associated with poor quality [20]. This may have biased our results towards the null if in fact these smaller facilities have lower levels of readiness and quality as we have hypothesized here. Having data on more lower level facilities in future data collection and analysis efforts may strengthen the findings of this association. Finally, direct observation requires data collectors to be present for the entire length of the service. For some services, such as antenatal care, this may be a relatively short consultation. However, for other services such as labor and delivery, the service can extend over the course of more than 24 hours which in many cases is impractical for data collectors. Record review of patient charts is an alternative to direct observation that has been used in high-income countries and has been proposed for use in LMICs. However, in many LMICs, individual patient charts are not readily available for most services, and both patient charts and registers in LMICs have been found to often be incomplete and of questionable quality [60–62]. While it is not impossible to collect provision of care data in LMICs, the resource-intensiveness and logistical challenges have limited the availability of provision of care data. Of particular concern, the countries with the poorest health indicators often do not have provision of care data and may be left behind if they are excluded from quality of care measurement and ultimately quality improvement efforts. However, many LMICs do have data on facility readiness. Our results suggest that these data can provide useful insights into the foundations of a health system which are required for delivering high quality care.

To our knowledge, this is the first study that has examined the client-level association between facility readiness and provision of care for ANC in LMICs. Despite our important findings, there are several limitations to this study. First, the analysis is limited in scope as it is comprised of one country in Latin America, one country in South-East Asia, two countries in East/Southern Africa, and one country in West Africa. Consequently, our findings may not be generalizable to LMICs globally but are likely generalizable to similar countries. Second, because we limited this analysis to first ANC consultations, it restricted the sample size for this study. However, the surveys in Haiti and Malawi were a census of facilities and the sample size for Tanzania was the largest of the surveys, thus resulting in a sufficient number of observations. Third, in Malawi and Nepal, the number of facilities with readiness less than 50 was small for certain index constructions (i.e., Nepal and Malawi expert simple n = 15). Our analysis in these countries and for these index constructions may be susceptible to outliers, potentially influencing our identification of an inflection point. Fourth, this analysis was limited to ANC services and may not be representative of primary health care services more broadly. Future research to investigate this association across the continuum of care for women and children would help to build evidence across the health system. Finally, while we controlled for individual, health worker, and facility-level characteristics, there is potential for residual uncontrolled confounding due to unmeasured covariates. However, the SPA collects a range of facility, health worker, and individual characteristics and the covariates selected are likely representative of the most influential factors.

## Conclusions

The results of this study suggest that facility readiness is not a proxy for provision of care, but that there is a statistically significant and meaningful association between facility readiness and provision of care, consistent with the relationship postulated in quality of care frameworks. Our results also suggest a minimum level of facility readiness is needed in order to deliver high quality ANC services in five LMICs. Collecting data on facility readiness is necessary for understanding the foundations of health systems and for quality improvement efforts particularly in countries with the lowest levels of service quality. However, a comprehensive view of quality of care should include both facility readiness and provision of care measures.

### Electronic supplementary material

Below is the link to the electronic supplementary material.

Supplementary Material 1: Additional File 1: This additional file contains supplementary text, tables, and figures referenced in the main manuscript.

## Data Availability

The SPA datasets analyzed during the current study are available in the DHS repository, [https://dhsprogram.com/data/available-datasets.cfm] [23].

## Abbreviations

ANC: antenatal care
DHS: Demographic and Health Surveys
EmONC: Emergency Obstetric and Newborn Care
HFA: health facility assessment
LMICs: low-income and middle-income countries
PCA: principal component analysis
QIQ: Quick Investigation of Quality
SARA: Service Availability and Readiness Assessment
SDG: Sustainable Development Goal
SPA: Service Provision Assessment
UHC: universal health coverage
