# Supplementary material for: Association between antenatal care facility readiness and provision of care at the client level and facility level in five low- and middle-income countries
Source: BMC Health Serv Res. 2023 Oct 17;23:1109. doi: 10.1186/s12913-023-10106-5 (PMC10583346; doi:10.1186/s12913-023-10106-5)
Supplement: Supplementary file 1 — Supplementary Material 1: Additional File 1: This additional file contains supplementary text, tables, and figures referenced in the main manuscript. [file 12913_2023_10106_MOESM1_ESM.pdf]

ADDITIONAL FILE 1

Supplementary Text

Physicians include: generalist medical doctor, specialist medical doctor, surgeon

Clinical officers include: medical officer, assistant medical officer, clinical officer, clinical technician, medical assistant, assistant clinical officer, senior technician

Nurses/Midwives include: registered nurse, registered nurse with diploma, nurse, community health nurse, nurse/midwife, registered nurse midwife, enrolled nurse, enrolled nurse midwife, enrolled midwife/nurse midwife technician, nurse assistant/attendant, midwife, auxiliary nurse, auxiliary nurse midwife, health assistant, other community health workers

Supplementary Table 1: Full Sample Size and Analytical Sample Size, Haiti, Malawi, Nepal, Senegal, and Tanzania

	Haiti	Malawi	Nepal	Senegal	Tanzania
Total number of facilities	905	977	926	371	1,188
Total number of facilities offering ANC	832	643	889	323	1,031
Total number of facilities offering ANC and with client observations	452	412	458	290	815
Total number of facilities offering ANC and with first visit ANC client observations	358	257	303	182	648
Total number of facilities offering ANC and with first visit ANC client observations which are complete cases	**358**	**253**	**282**	**179**	**632**
Total number of ANC client observations	1,620	2,068	1,509	849	4,007
Total number of first visit ANC client observations	785	859	573	307	1,754
Total number of first visit ANC client observations which are complete cases	**779**	**815**	**520**	**297**	**1,681**

Supplementary Table 2: Comparison of All Cases and Complete Cases for Key Background Characteristics, Haiti, Malawi, Nepal, Senegal, and Tanzania

	Haiti			Malawi			Nepal			Senegal			Tanzania		
	All cases	Complete cases	p-value	All cases	Complete cases	p-value	All cases	Complete cases	p-value	All cases	Complete cases	p-value	All cases	Complete cases	p-value
**Facility characteristics**	N = 358	N = 358		N = 257	N = 253		N = 303	N = 282		N = 182	N = 179		N = 648	N = 632	
Facility type			1			0.995			0.903			0.995			0.997
Hospital	19.6%	19.6%		25.3%	24.9%		41.3%	42.9%		9.3%	9.5%		26.7%	26.6%	
Health center/clinic	51.7%	51.7%		72.4%	72.7%		33.7%	33.3%		18.1%	18.4%		38.9%	39.1%	
Dispensary	28.8%	28.8%		2.3%	2.4%		25.1%	23.8%		72.5%	72.1%		34.4%	34.3%	
Managing authority			1			0.975			1			1			0.986
Government	45.3%	45.3%		70.8%	71.5%		84.2%	84.0%		91.2%	91.6%		73.8%	73.6%	
Private non-faith based	36.0%	36.0%		6.2%	6.3%		15.2%	15.2%		8.8%	8.4%		6.9%	6.8%	
Private faith based	18.7%	18.7%		23.0%	22.1%		0.7%	0.7%		--	--		19.3%	19.6%	
Urban/rural			1			1			--			1			1
Urban	45.3%	45.3%		20.2%	20.2%		--	--		46.2%	46.4%		32.7%	32.6%	
Rural	54.7%	54.7%		79.8%	79.8%		--	--		53.8%	53.6%		67.3%	67.4%	
Average number of staff	12.6	12.6	1	7.8	7.7	0.957	10.2	10.5	0.668	7.64	7.70	0.929	10.4	10.4	0.995
**Health worker characteristics**	N = 406	N = 406		N = 288	N = 283		N = 361	N = 337		N = 187	N = 184		N = 823	N = 799	
Qualification			1			1			0.977			1			0.989
Physician	42.4%	42.4%		0.4%	0.0%		15.8%	16.3%		1.1%	1.1%		1.8%	1.8%	
Clinical officer	0.0%	0.0%		5.2%	5.0%		5.5%	5.3%		11.2%	11.4%		3.0%	3.1%	
Nurse/Midwife	57.6%	57.6%		94.4%	95.1%		78.7%	78.3%		87.7%	87.5%		95.1%	95.1%	
Gender			1			1			0.97			1			0.945
Male	32.5%	32.5%		25.0%	25.1%		11.4%	11.0%		9.6%	9.8%		12.0%	12.3%	
Female	67.5%	67.5%		75.0%	74.9%		88.6%	89.0%		90.4%	90.2%		88.0%	87.7%	
**Individual characteristics**	N = 785	N = 779		N = 859	N = 815		N = 573	N = 520		N = 307	N = 297		N = 1754	N = 1681	
Number of weeks pregnant	--	--	--	21.3	21.3	0.855	19.7	19.7	0.963	--	--	--	22.4	22.4	0.991
Client had a previous pregnancy	33.2%	33.4%	1	24.4%	24.9%	0.871	47.5%	48.5%	0.789	24.1%	24.2%	1	26.0%	25.7%	0.871
Age (years)	26.6	26.6	1	25.1	25.1	0.946	22.9	22.9	0.998	25.8	25.8	1	25.8	25.9	0.888

	Haiti			Malawi			Nepal			Senegal			Tanzania		
	**All cases**	**Complete cases**	**p-value**	**All cases**	**Complete cases**	**p-value**	**All cases**	**Complete cases**	**p-value**	**All cases**	**Complete cases**	**p-value**	**All cases**	**Complete cases**	**p-value**
Highest level of education attained			0.997			0.888			0.878			0.993			0.948
Never attended school	14.5%	14.1%		15.0%	13.7%		26.7%	25.0%		53.7%	52.5%		19.3%	18.5%	
Primary	39.7%	39.9%		63.4%	64.2%		8.9%	8.3%		25.1%	25.6%		59.5%	60.1%	
Secondary	43.4%	43.6%		19.3%	20.0%		46.1%	48.1%		19.2%	19.9%		19.0%	19.1%	
Higher	2.3%	2.3%		2.2%	2.1%		18.3%	18.7%		2.0%	2.0%		2.2%	2.3%	

Supplementary Table 3: Number of Beds and Number of Staff by Facility Type, Haiti, Malawi, Nepal, Senegal, and Tanzania

Facility type	Number of staff	Number of beds
**Haiti**		
Hospital	27.0	62.5
Health center with beds	15.8	9.0
Health center without beds	11.2	1.3
Dispensary	4.8	0.7
**Malawi**		
Hospital	16.2	150.1
Health center	5.9	4.3
Dispensary	5.5	0.4
Clinic	3.7	2.1
**Nepal**		
Hospital	18.0	153.8
Health center	7.5	2.2
Health post	4.7	0.1
Sub-health post	3.5	0.1
**Senegal**		
Hospital	19.5	59.6
Health center	12.9	16.7
Clinic	5.3	4.4
**Tanzania**		
Hospital	25.2	148.4
Health center	8.0	15.7
Clinic	4.2	1.9
Dispensary	3.2	2.0

Supplementary Figure 1: Distribution of Facility Readiness Scores, Haiti, Malawi, Nepal, Senegal, and Tanzania

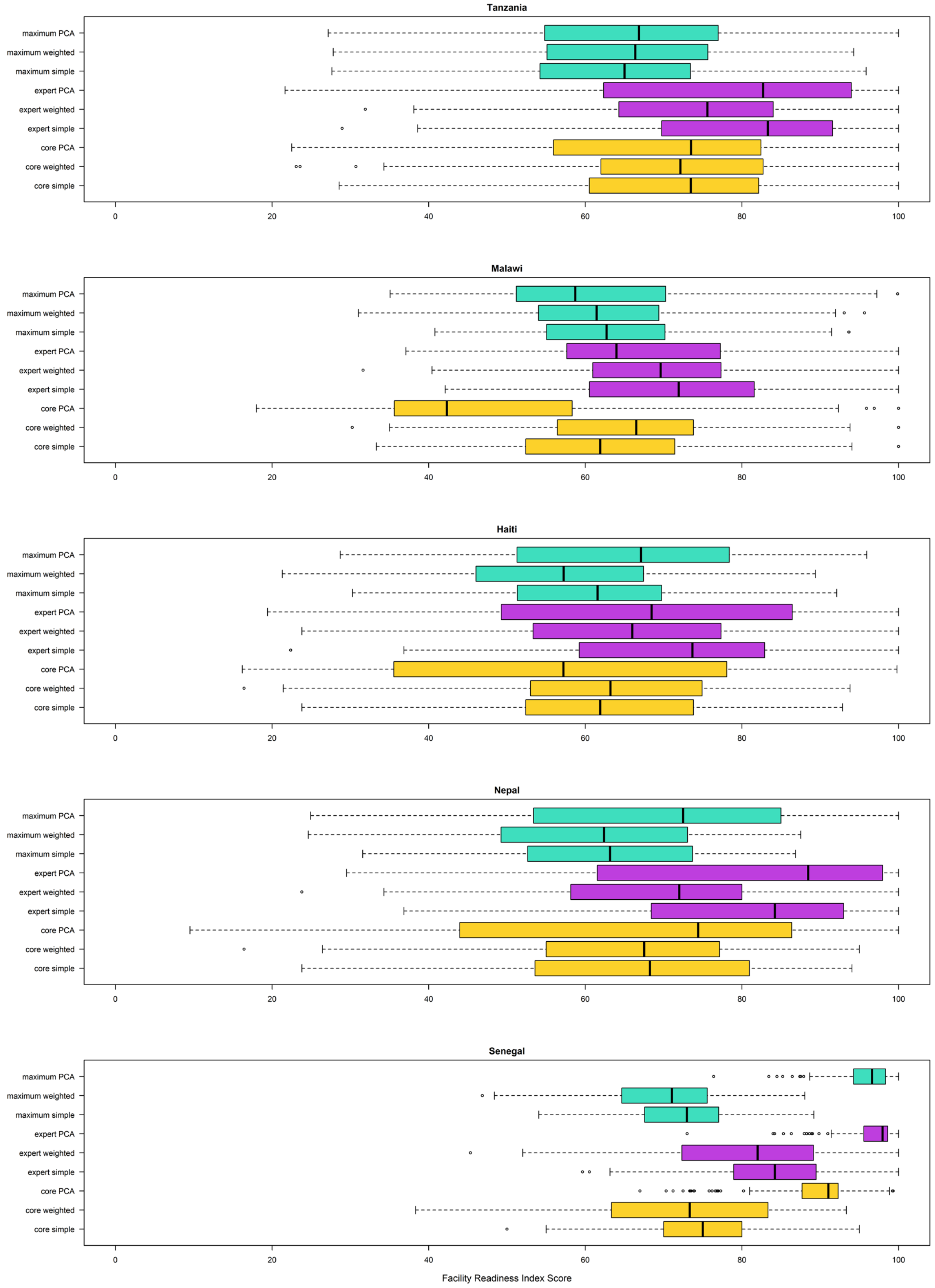

Supplementary Figure 2: Distribution of Provision of Care Scores, Haiti, Malawi, Nepal, Senegal, and Tanzania

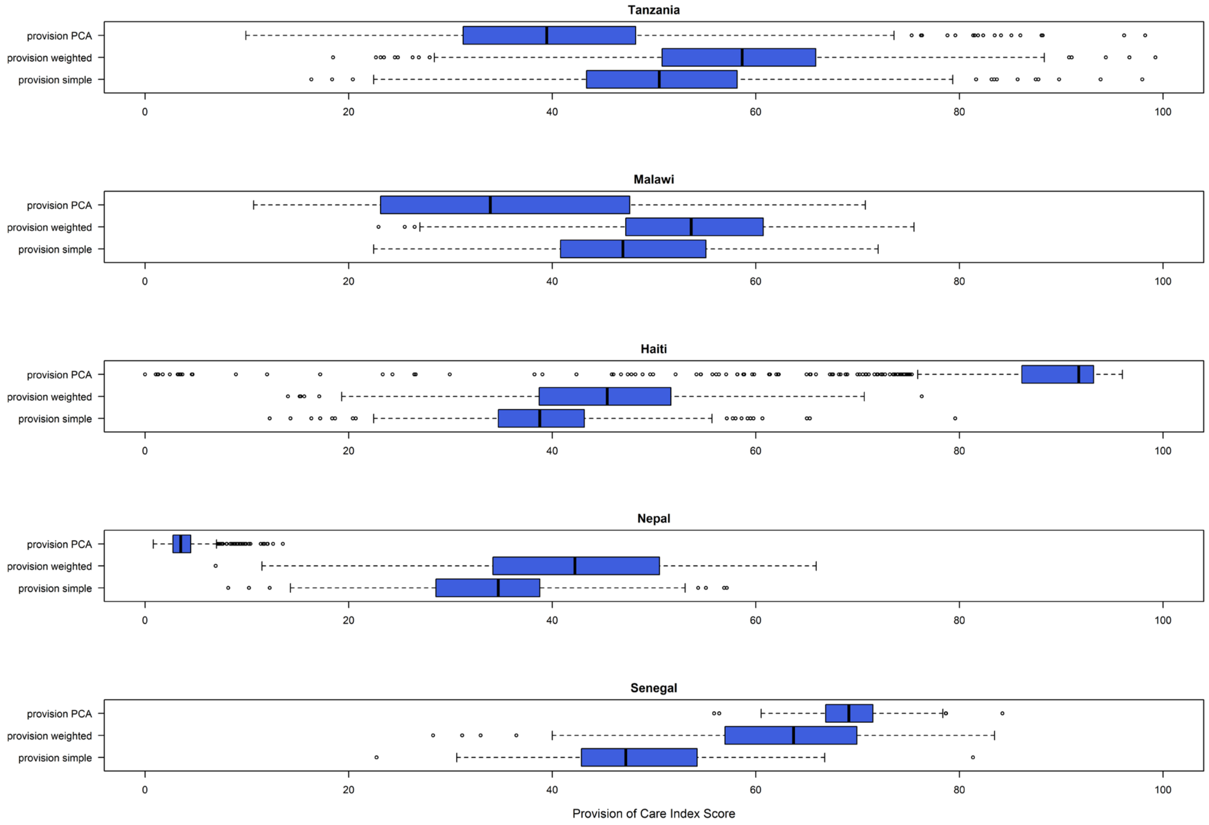

Supplementary Table 4: Bivariate Association between Provision of Care and Facility Readiness, Haiti, Malawi, Nepal, Senegal, and Tanzania

Haiti			
	Estimate	Std. Error	p-value
Core simple	0.044	0.034	0.195
Core weighted	0.033	0.032	0.302
Core PCA	-0.005	0.022	0.834
Expert simple	0.019	0.031	0.540
Expert weighted	0.019	0.029	0.510
Expert PCA	-0.001	0.023	0.969
Maximum simple	0.044	0.038	0.251
Maximum weighted	0.025	0.034	0.459
Maximum PCA	-0.007	0.030	0.824
**Malawi**			
	Estimate	Std. Error	p-value
Core simple	0.182	0.042	0.000
Core weighted	0.129	0.042	0.003
Core PCA	0.125	0.031	0.000
Expert simple	0.211	0.043	0.000
Expert weighted	0.198	0.043	0.000
Expert PCA	0.170	0.037	0.000
Maximum simple	0.206	0.049	0.000
Maximum weighted	0.204	0.048	0.000
Maximum PCA	0.174	0.040	0.000
**Nepal**			
	Estimate	Std. Error	p-value
Core simple	-0.012	0.041	0.766
Core weighted	0.005	0.042	0.897
Core PCA	-0.045	0.029	0.121
Expert simple	-0.014	0.042	0.749
Expert weighted	-0.002	0.047	0.964
Expert PCA	-0.039	0.033	0.237
Maximum simple	-0.027	0.049	0.581
Maximum weighted	-0.009	0.045	0.841
Maximum PCA	-0.044	0.035	0.212
**Senegal**			
	Estimate	Std. Error	p-value
Core simple	0.140	0.075	0.063
Core weighted	0.107	0.052	0.042
Core PCA	0.082	0.101	0.417
Expert simple	0.087	0.078	0.266
Expert weighted	0.049	0.060	0.417
Expert PCA	0.052	0.185	0.780
Maximum simple	0.193	0.087	0.028
Maximum weighted	0.088	0.078	0.258
Maximum PCA	0.183	0.185	0.325

Tanzania			
	Estimate	Std. Error	p-value
Core simple	0.204	0.027	0.000
Core weighted	0.160	0.026	0.000
Core PCA	0.170	0.024	0.000
Expert simple	0.170	0.026	0.000
Expert weighted	0.178	0.027	0.000
Expert PCA	0.130	0.022	0.000
Maximum simple	0.181	0.030	0.000
Maximum weighted	0.189	0.028	0.000
Maximum PCA	0.162	0.027	0.000

Supplementary Figure 3: Bivariate Association between Provision of Care and Facility Readiness, Haiti

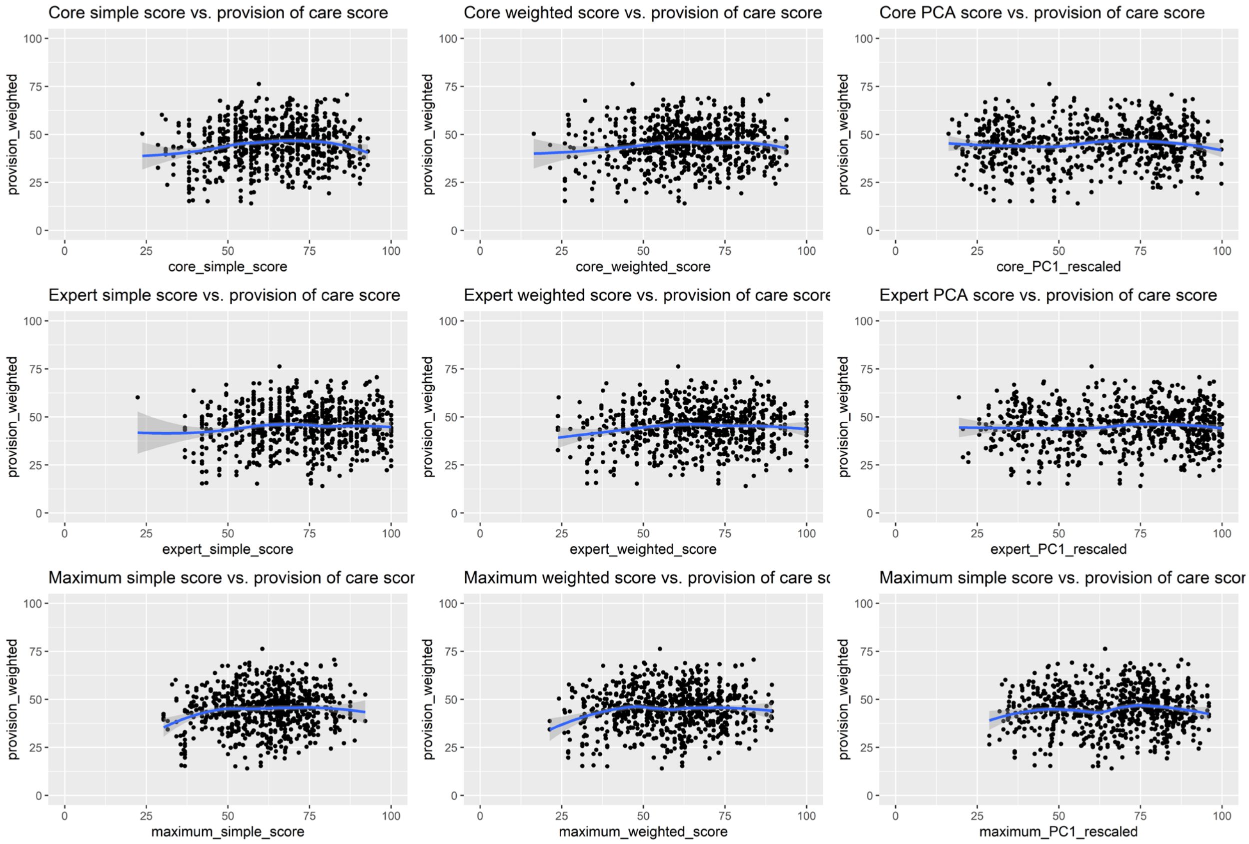

Supplementary Figure 4: Bivariate Association between Provision of Care and Facility Readiness, Malawi

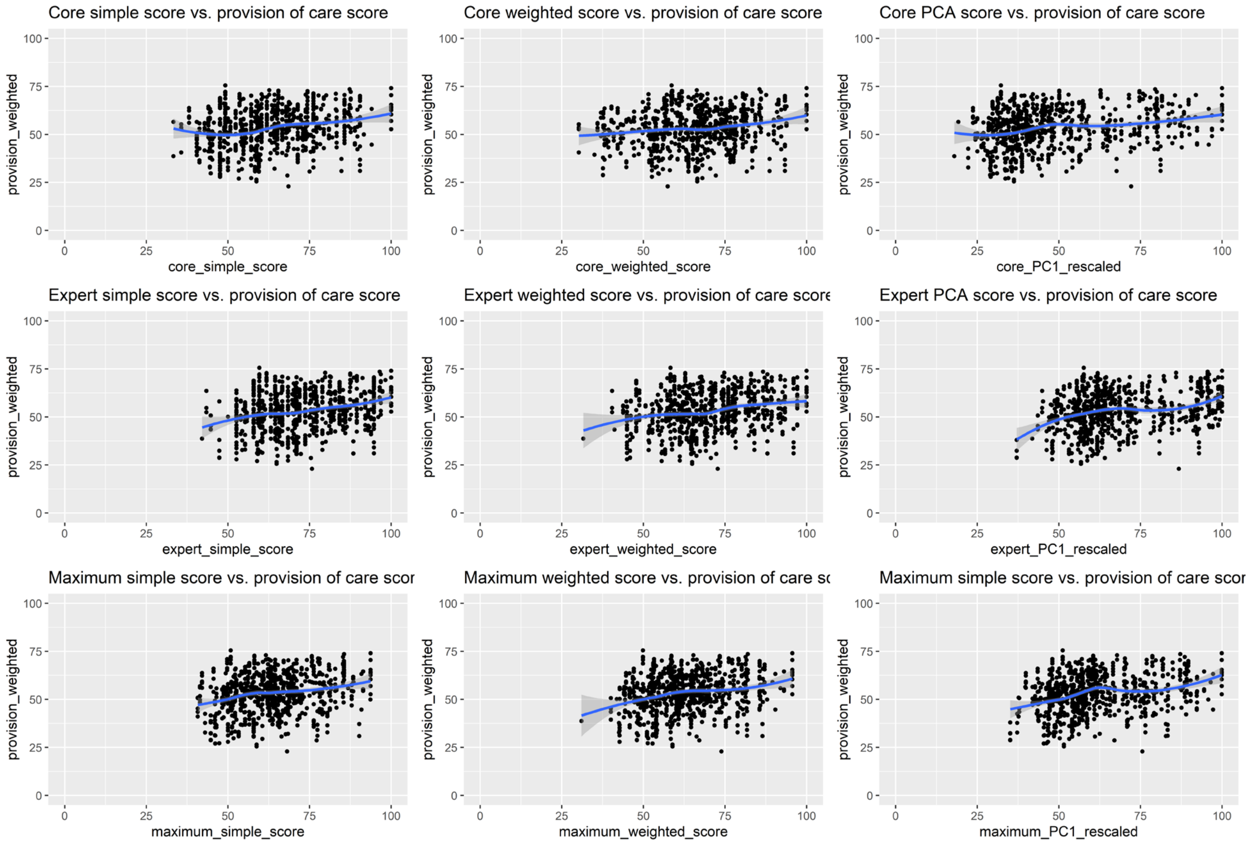

Supplementary Figure 5: Bivariate Association between Provision of Care and Facility Readiness, Nepal

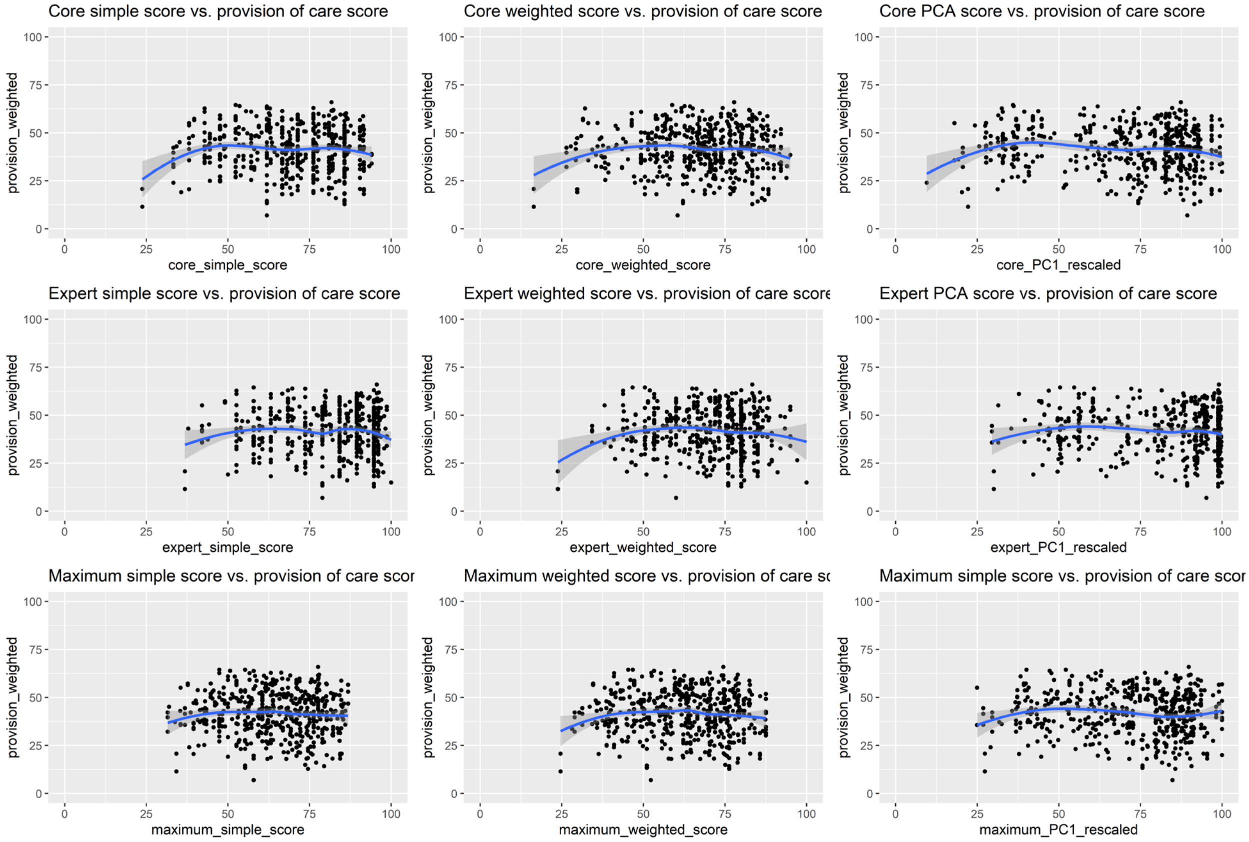

Supplementary Figure 6: Bivariate Association between Provision of Care and Facility Readiness, Senegal

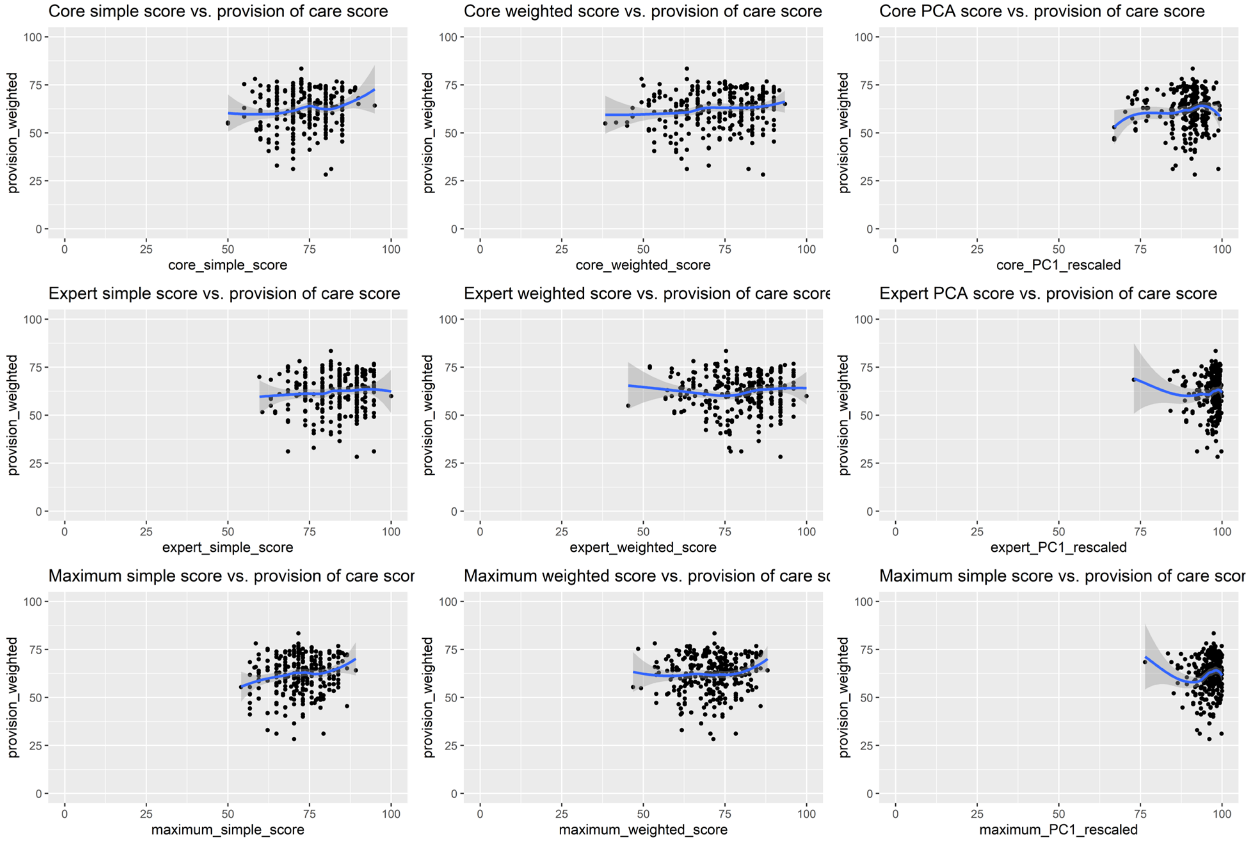

Supplementary Figure 7: Bivariate Association between Provision of Care and Facility Readiness, Tanzania

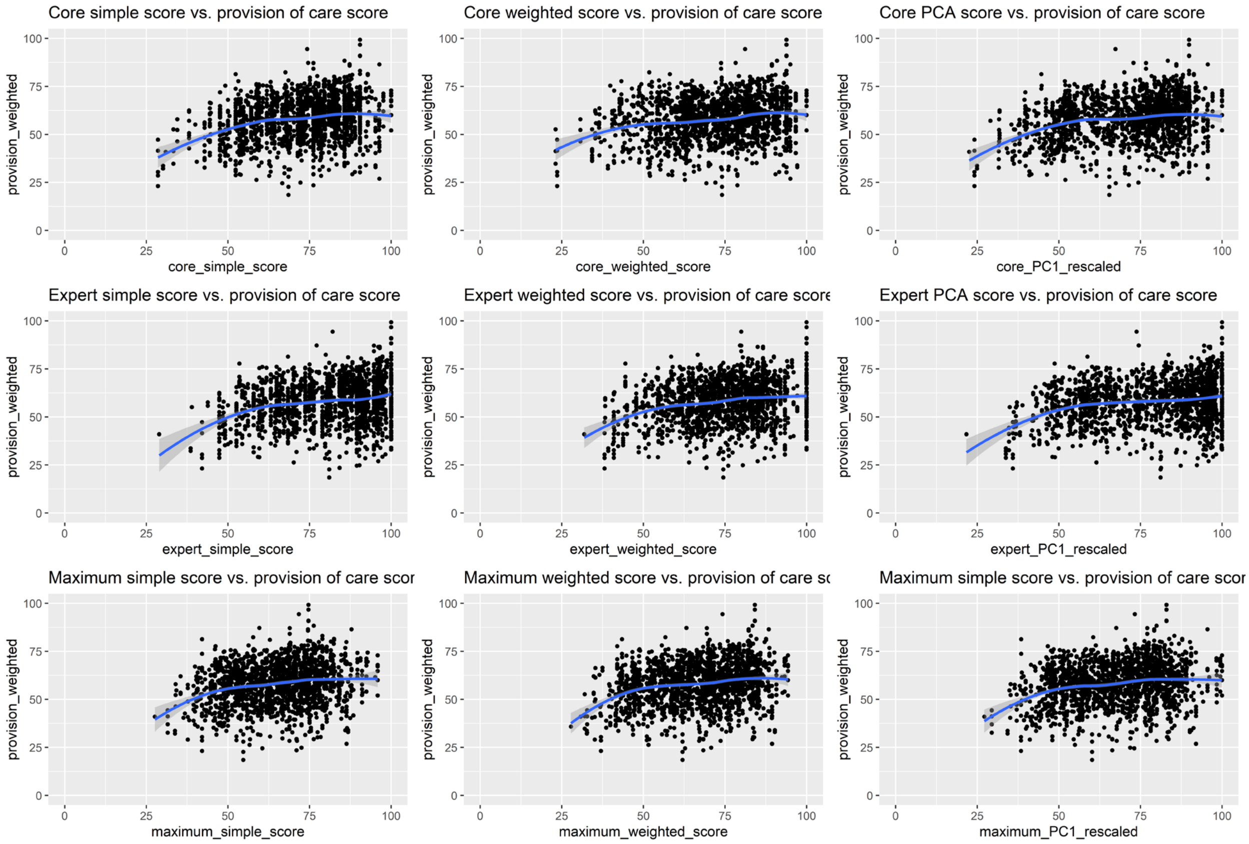

Supplementary Table 5: Facility Level - Bivariate Association between Provision of Care and Facility Readiness, Haiti, Malawi, Nepal, Senegal, and Tanzania

Haiti			
	Estimate	Std. Error	p-value
Core simple	0.031	0.034	0.360
Core weighted	0.022	0.031	0.490
Core PCA	-0.014	0.022	0.526
Expert simple	0.007	0.031	0.816
Expert weighted	0.009	0.029	0.757
Expert PCA	-0.010	0.023	0.656
Maximum simple	0.032	0.038	0.409
Maximum weighted	0.013	0.034	0.697
Maximum PCA	-0.018	0.030	0.545
**Malawi**			
	Estimate	Std. Error	p-value
Core simple	0.185	0.042	0.000
Core weighted	0.134	0.043	0.002
Core PCA	0.126	0.031	0.000
Expert simple	0.216	0.044	0.000
Expert weighted	0.206	0.043	0.000
Expert PCA	0.172	0.038	0.000
Maximum simple	0.210	0.050	0.000
Maximum weighted	0.208	0.049	0.000
Maximum PCA	0.175	0.041	0.000
**Nepal**			
	Estimate	Std. Error	p-value
Core simple	-0.014	0.042	0.732
Core weighted	0.005	0.043	0.901
Core PCA	-0.044	0.029	0.131
Expert simple	-0.015	0.043	0.730
Expert weighted	-0.004	0.047	0.938
Expert PCA	-0.039	0.034	0.244
Maximum simple	-0.033	0.049	0.500
Maximum weighted	-0.013	0.045	0.773
Maximum PCA	-0.047	0.036	0.195
**Senegal**			
	Estimate	Std. Error	p-value
Core simple	0.111	0.073	0.131
Core weighted	0.101	0.051	0.050
Core PCA	0.054	0.099	0.586
Expert simple	0.080	0.076	0.295
Expert weighted	0.050	0.058	0.391
Expert PCA	0.007	0.173	0.970
Maximum simple	0.169	0.085	0.049
Maximum weighted	0.075	0.075	0.320
Maximum PCA	0.130	0.176	0.463

Tanzania			
	Estimate	Std. Error	p-value
Core simple	0.208	0.028	0.000
Core weighted	0.165	0.027	0.000
Core PCA	0.172	0.024	0.000
Expert simple	0.171	0.027	0.000
Expert weighted	0.182	0.027	0.000
Expert PCA	0.131	0.022	0.000
Maximum simple	0.188	0.031	0.000
Maximum weighted	0.195	0.029	0.000
Maximum PCA	0.167	0.028	0.000

Supplementary Figure 8: Facility Level - Bivariate Association between Provision of Care and Facility Readiness, Haiti

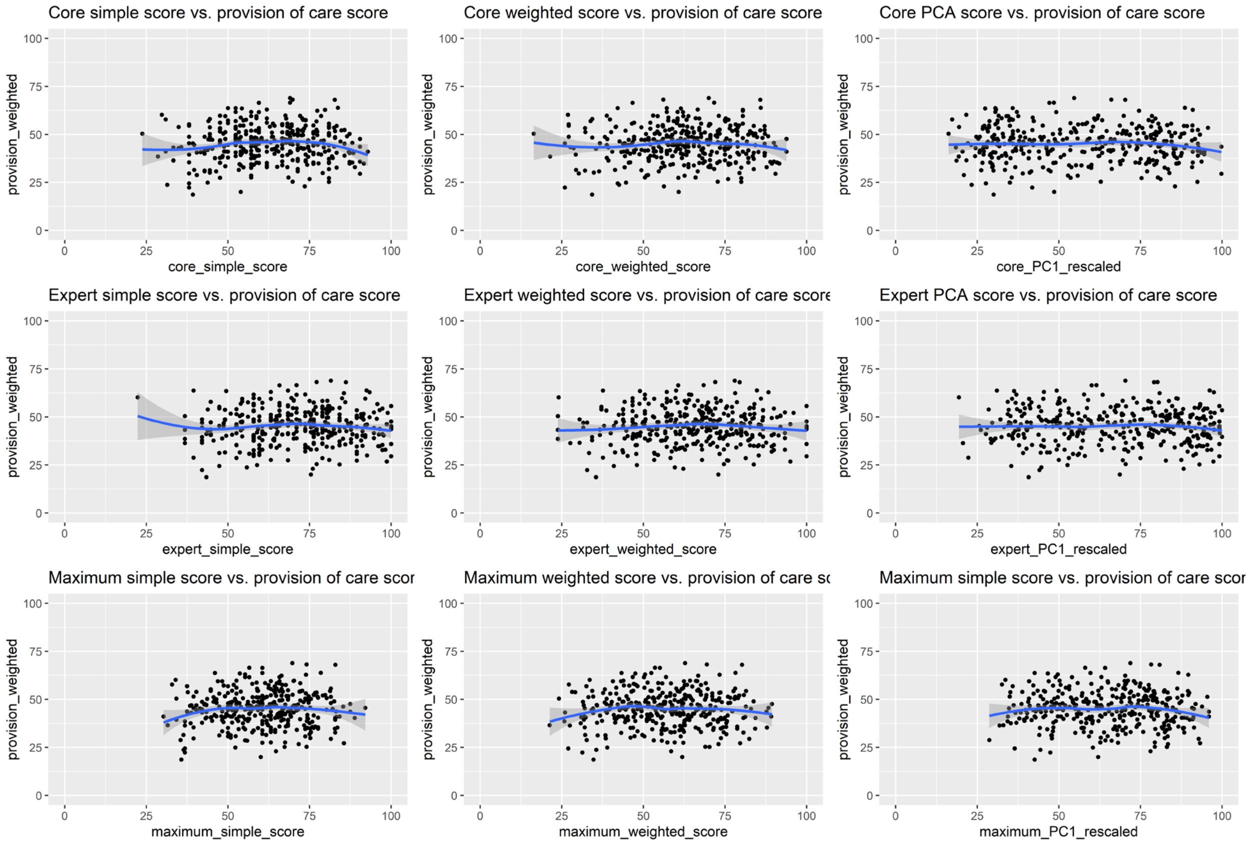

Supplementary Figure 9: Facility Level - Bivariate Association between Provision of Care and Facility Readiness, Malawi

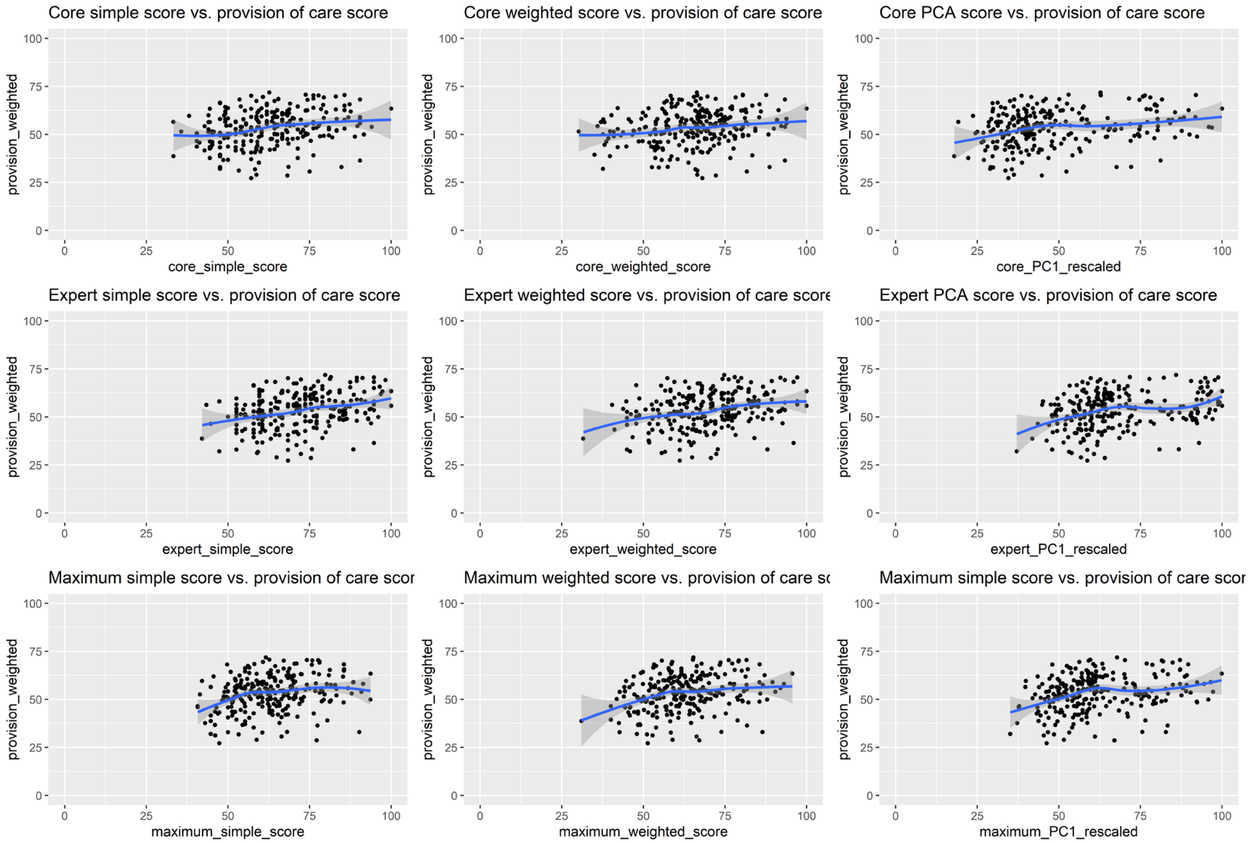

Supplementary Figure 10: Facility Level - Bivariate Association between Provision of Care and Facility Readiness, Nepal

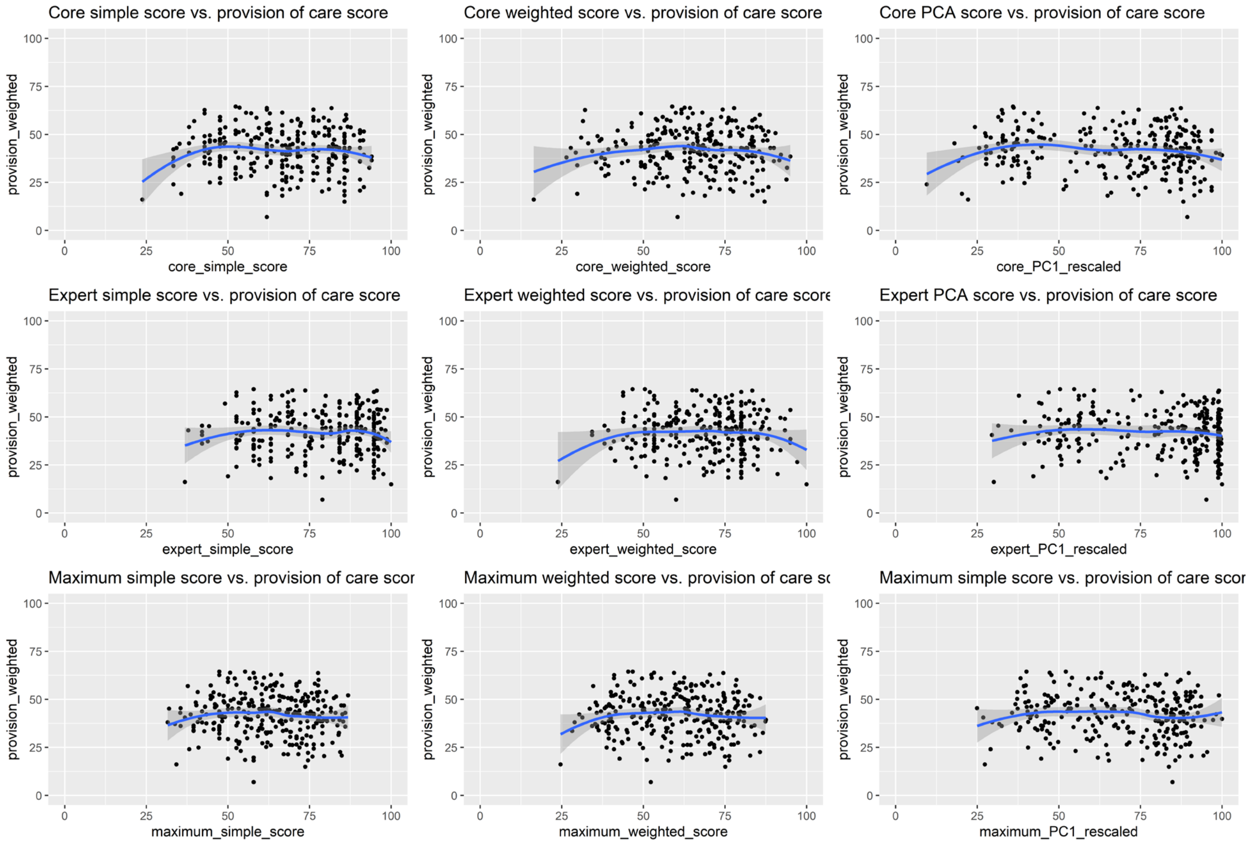

Supplementary Figure 11: Facility Level - Bivariate Association between Provision of Care and Facility Readiness, Senegal

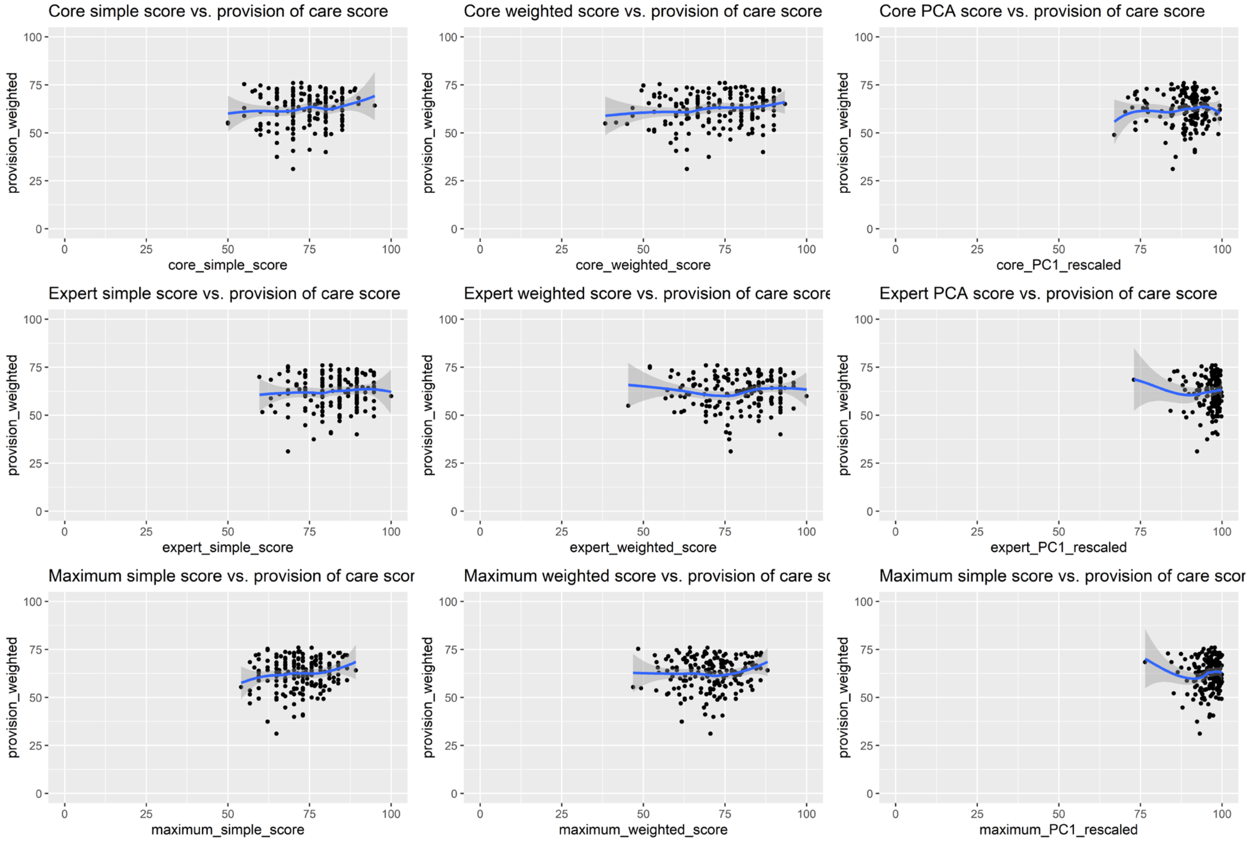

Supplementary Figure 12: Facility Level - Bivariate Association between Provision of Care and Facility Readiness, Tanzania

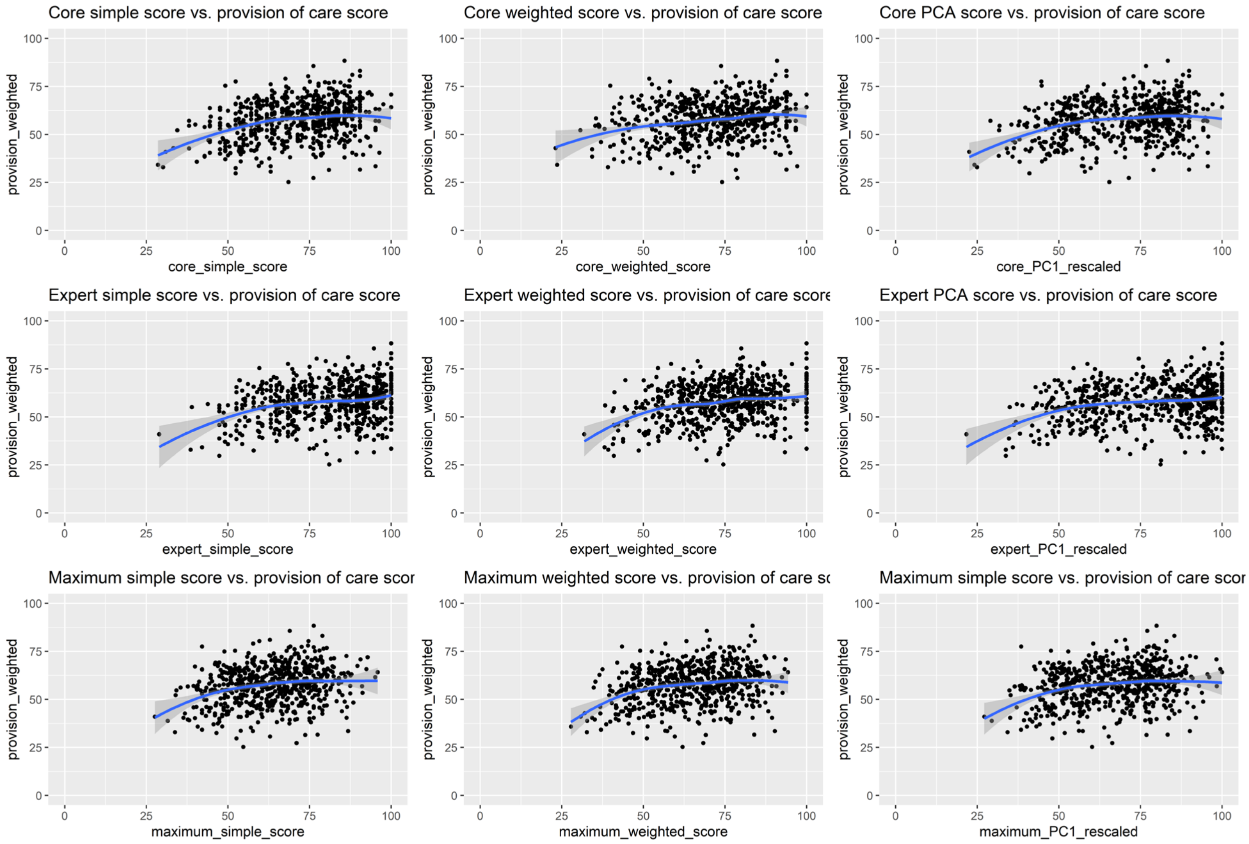

Supplementary Table 6: Client Level Comparison of Index Estimates, Haiti, Malawi, Nepal, Senegal, and Tanzania

Haiti				
	Mean difference	Std. Deviation	p-value	Mean difference / Std. Deviation
core simple v. core weighted	0.056	0.017	0.001*	3.365
core simple v. core PCA	0.069	0.019	0.000*	3.588
core simple v. expert simple	0.033	0.016	0.042	2.030
core simple v. expert weighted	0.038	0.019	0.049	1.965
core simple v. expert PCA	0.064	0.021	0.002	3.046
core simple v. maximum simple	-0.003	0.019	0.886	-0.143
core simple v. maximum weighted	0.020	0.016	0.197	1.289
core simple v. maximum PCA	0.051	0.020	0.010	2.578
core weighted v. core PCA	0.013	0.023	0.585	0.546
core weighted v. expert simple	-0.023	0.019	0.215	-1.239
core weighted v. expert weighted	-0.019	0.016	0.239	-1.177
core weighted v. expert PCA	0.007	0.023	0.752	0.315
core weighted v. maximum simple	-0.059	0.024	0.016	-2.407
core weighted v. maximum weighted	-0.036	0.019	0.053	-1.934
core weighted v. maximum PCA	-0.006	0.026	0.829	-0.216
core PCA v. expert simple	-0.036	0.018	0.053	-1.937
core PCA v. expert weighted	-0.031	0.020	0.116	-1.571
core PCA v. expert PCA	-0.005	0.009	0.562	-0.580
core PCA v. maximum simple	-0.071	0.028	0.010	-2.586
core PCA v. maximum weighted	-0.048	0.022	0.031	-2.159
core PCA v. maximum PCA	-0.018	0.018	0.316	-1.002
expert simple v. expert weighted	0.005	0.011	0.674	0.421
expert simple v. expert PCA	0.031	0.014	0.031	2.156
expert simple v. maximum simple	-0.036	0.020	0.075	-1.780
expert simple v. maximum weighted	-0.013	0.017	0.451	-0.753
expert simple v. maximum PCA	0.018	0.017	0.287	1.064
expert weighted v. expert PCA	0.026	0.017	0.125	1.535
expert weighted v. maximum simple	-0.040	0.023	0.083	-1.732
expert weighted v. maximum weighted	-0.017	0.017	0.298	-1.041
expert weighted v. maximum PCA	0.013	0.021	0.528	0.631
expert PCA v. maximum simple	-0.066	0.026	0.011	-2.545
expert PCA v. maximum weighted	-0.043	0.021	0.043	-2.028
expert PCA v. maximum PCA	-0.043	0.021	0.043	-2.028
maximum simple v. maximum weighted	0.023	0.013	0.076	1.772
maximum simple v. maximum PCA	0.053	0.016	0.001*	3.321
maximum weighted v. maximum PCA	0.030	0.016	0.055	1.922
**Malawi**				
	Mean difference	Std. Deviation	p-value	Mean difference / Std. Deviation
core simple v. core weighted	0.100	0.031	0.001*	3.260
core simple v. core PCA	0.032	0.026	0.218	1.232
core simple v. expert simple	-0.048	0.031	0.118	-1.563
core simple v. expert weighted	-0.012	0.033	0.720	-0.358
core simple v. expert PCA	-0.038	0.039	0.331	-0.971
core simple v. maximum simple	-0.027	0.036	0.444	-0.766
core simple v. maximum weighted	-0.048	0.031	0.124	-1.537
core simple v. maximum PCA	-0.028	0.037	0.454	-0.749
core weighted v. core PCA	-0.069	0.041	0.097	-1.657
core weighted v. expert simple	-0.149	0.038	0.000*	-3.937
core weighted v. expert weighted	-0.112	0.031	0.000*	-3.616
core weighted v. expert PCA	-0.138	0.048	0.004	-2.866
core weighted v. maximum simple	-0.128	0.047	0.007	-2.693
core weighted v. maximum weighted	-0.148	0.043	0.001*	-3.465
core weighted v. maximum PCA	-0.128	0.052	0.014	-2.456
core PCA v. expert simple	-0.080	0.031	0.010	-2.588

core PCA v. expert weighted	-0.044	0.034	0.195	-1.297
core PCA v. expert PCA	-0.070	0.023	0.003	-2.979
core PCA v. maximum simple	-0.059	0.041	0.150	-1.438
core PCA v. maximum weighted	-0.080	0.035	0.024	-2.254
core PCA v. maximum PCA	-0.060	0.029	0.039	-2.063
expert simple v. expert weighted	0.037	0.024	0.125	1.533
expert simple v. expert PCA	0.010	0.025	0.675	0.419
expert simple v. maximum simple	0.021	0.038	0.588	0.542
expert simple v. maximum weighted	0.001	0.037	0.989	0.014
expert simple v. maximum PCA	0.021	0.038	0.593	0.534
expert weighted v. expert PCA	-0.026	0.033	0.432	-0.785
expert weighted v. maximum simple	-0.016	0.044	0.719	-0.360
expert weighted v. maximum weighted	-0.036	0.037	0.325	-0.985
expert weighted v. maximum PCA	-0.016	0.043	0.711	-0.371
expert PCA v. maximum simple	0.011	0.045	0.814	0.236
expert PCA v. maximum weighted	-0.010	0.040	0.806	-0.246
expert PCA v. maximum PCA	-0.010	0.040	0.806	-0.246
maximum simple v. maximum weighted	-0.020	0.028	0.468	-0.725
maximum simple v. maximum PCA	0.000	0.028	0.993	-0.008
maximum weighted v. maximum PCA	0.020	0.033	0.541	0.611
**Nepal**				
	Mean difference	Std. Deviation	p-value	Mean difference / Std. Deviation
core simple v. core weighted	0.017	0.020	0.414	0.817
core simple v. core PCA	0.051	0.024	0.031	2.153
core simple v. expert simple	0.000	0.023	0.997	-0.004
core simple v. expert weighted	0.037	0.030	0.211	1.250
core simple v. expert PCA	0.046	0.029	0.107	1.613
core simple v. maximum simple	-0.046	0.026	0.074	-1.788
core simple v. maximum weighted	-0.026	0.019	0.182	-1.334
core simple v. maximum PCA	-0.033	0.029	0.260	-1.127
core weighted v. core PCA	0.035	0.033	0.291	1.056
core weighted v. expert simple	-0.017	0.029	0.571	-0.567
core weighted v. expert weighted	0.020	0.032	0.520	0.644
core weighted v. expert PCA	0.029	0.036	0.408	0.827
core weighted v. maximum simple	-0.063	0.035	0.074	-1.787
core weighted v. maximum weighted	-0.043	0.026	0.102	-1.635
core weighted v. maximum PCA	-0.050	0.039	0.204	-1.270
core PCA v. expert simple	-0.052	0.026	0.046	-1.997
core PCA v. expert weighted	-0.015	0.036	0.684	-0.407
core PCA v. expert PCA	-0.005	0.016	0.743	-0.327
core PCA v. maximum simple	-0.098	0.036	0.006	-2.737
core PCA v. maximum weighted	-0.077	0.034	0.024	-2.250
core PCA v. maximum PCA	-0.085	0.024	0.000*	-3.520
expert simple v. expert weighted	0.037	0.021	0.072	1.798
expert simple v. expert PCA	0.046	0.018	0.011	2.547
expert simple v. maximum simple	-0.046	0.034	0.177	-1.349
expert simple v. maximum weighted	-0.026	0.027	0.342	-0.950
expert simple v. maximum PCA	-0.033	0.029	0.262	-1.122
expert weighted v. expert PCA	0.009	0.031	0.768	0.296
expert weighted v. maximum simple	-0.083	0.043	0.053	-1.936
expert weighted v. maximum weighted	-0.063	0.032	0.050	-1.964
expert weighted v. maximum PCA	-0.070	0.042	0.093	-1.681
expert PCA v. maximum simple	-0.092	0.038	0.016	-2.402
expert PCA v. maximum weighted	-0.072	0.035	0.039	-2.063
expert PCA v. maximum PCA	-0.072	0.035	0.039	-2.063
maximum simple v. maximum weighted	0.020	0.020	0.322	0.990
maximum simple v. maximum PCA	0.013	0.026	0.613	0.506
maximum weighted v. maximum PCA	-0.007	0.032	0.823	-0.223
**Senegal**				

	Mean difference	Std. Deviation	p-value	Mean difference / Std. Deviation
core simple v. core weighted	0.046	0.037	0.212	1.247
core simple v. core PCA	-0.008	0.065	0.896	-0.131
core simple v. expert simple	0.024	0.037	0.525	0.636
core simple v. expert weighted	0.099	0.040	0.013	2.498
core simple v. expert PCA	0.036	0.147	0.809	0.242
core simple v. maximum simple	-0.107	0.040	0.008	-2.652
core simple v. maximum weighted	0.029	0.032	0.369	0.898
core simple v. maximum PCA	-0.217	0.180	0.228	-1.207
core weighted v. core PCA	-0.054	0.081	0.502	-0.671
core weighted v. expert simple	-0.022	0.046	0.630	-0.482
core weighted v. expert weighted	0.053	0.036	0.139	1.478
core weighted v. expert PCA	-0.010	0.156	0.949	-0.064
core weighted v. maximum simple	-0.153	0.049	0.002	-3.091
core weighted v. maximum weighted	-0.017	0.047	0.717	-0.362
core weighted v. maximum PCA	-0.262	0.190	0.167	-1.380
core PCA v. expert simple	0.032	0.059	0.582	0.550
core PCA v. expert weighted	0.107	0.083	0.193	1.301
core PCA v. expert PCA	0.044	0.120	0.713	0.368
core PCA v. maximum simple	-0.098	0.077	0.199	-1.286
core PCA v. maximum weighted	0.037	0.079	0.636	0.473
core PCA v. maximum PCA	-0.208	0.156	0.182	-1.335
expert simple v. expert weighted	0.075	0.044	0.084	1.728
expert simple v. expert PCA	0.012	0.130	0.927	0.092
expert simple v. maximum simple	-0.131	0.053	0.013	-2.487
expert simple v. maximum weighted	0.005	0.052	0.924	0.095
expert simple v. maximum PCA	-0.240	0.172	0.162	-1.398
expert weighted v. expert PCA	-0.063	0.155	0.682	-0.409
expert weighted v. maximum simple	-0.206	0.059	0.001*	-3.460
expert weighted v. maximum weighted	-0.070	0.045	0.122	-1.546
expert weighted v. maximum PCA	-0.316	0.194	0.104	-1.625
expert PCA v. maximum simple	-0.143	0.148	0.336	-0.961
expert PCA v. maximum weighted	-0.007	0.153	0.964	-0.046
expert PCA v. maximum PCA	-0.007	0.153	0.964	-0.046
maximum simple v. maximum weighted	0.136	0.038	0.000*	3.613
maximum simple v. maximum PCA	-0.110	0.172	0.523	-0.639
maximum weighted v. maximum PCA	-0.245	0.183	0.180	-1.341
**Tanzania**				
	Mean difference	Std. Deviation	p-value	Mean difference / Std. Deviation
core simple v. core weighted	0.072	0.014	0.000*	4.963
core simple v. core PCA	0.035	0.008	0.000*	4.343
core simple v. expert simple	0.047	0.017	0.005	2.787
core simple v. expert weighted	0.032	0.018	0.081	1.744
core simple v. expert PCA	0.094	0.017	0.000*	5.408
core simple v. maximum simple	0.031	0.019	0.106	1.615
core simple v. maximum weighted	0.005	0.014	0.695	0.392
core simple v. maximum PCA	0.039	0.019	0.037	2.087
core weighted v. core PCA	-0.036	0.018	0.049	-1.967
core weighted v. expert simple	-0.025	0.021	0.235	-1.187
core weighted v. expert weighted	-0.040	0.020	0.046	-1.993
core weighted v. expert PCA	0.022	0.023	0.324	0.986
core weighted v. maximum simple	-0.040	0.024	0.094	-1.674
core weighted v. maximum weighted	-0.066	0.019	0.000*	-3.515
core weighted v. maximum PCA	-0.033	0.025	0.193	-1.303
core PCA v. expert simple	0.011	0.016	0.466	0.729
core PCA v. expert weighted	-0.003	0.018	0.848	-0.192
core PCA v. expert PCA	0.059	0.013	0.000*	4.569
core PCA v. maximum simple	-0.004	0.020	0.832	-0.212
core PCA v. maximum weighted	-0.030	0.016	0.055	-1.920

core PCA v. maximum PCA	0.003	0.017	0.836	0.207
expert simple v. expert weighted	-0.015	0.013	0.248	-1.156
expert simple v. expert PCA	0.047	0.009	0.000*	5.255
expert simple v. maximum simple	-0.016	0.020	0.432	-0.787
expert simple v. maximum weighted	-0.041	0.018	0.026	-2.231
expert simple v. maximum PCA	-0.008	0.018	0.658	-0.443
expert weighted v. expert PCA	0.062	0.016	0.000*	3.849
expert weighted v. maximum simple	-0.001	0.023	0.975	-0.031
expert weighted v. maximum weighted	-0.026	0.018	0.150	-1.441
expert weighted v. maximum PCA	0.007	0.021	0.744	0.327
expert PCA v. maximum simple	-0.063	0.021	0.002	-3.039
expert PCA v. maximum weighted	-0.088	0.019	0.000*	-4.571
expert PCA v. maximum PCA	-0.088	0.019	0.000*	-4.571
maximum simple v. maximum weighted	-0.026	0.013	0.043	-2.022
maximum simple v. maximum PCA	0.008	0.011	0.504	0.669
maximum weighted v. maximum PCA	0.033	0.014	0.017	2.376

* p<=0.0014 (Bonferroni correction for multiple comparisons)

Supplementary Table 7: Client Level Comparison of Index Estimates with Spline, Haiti, Malawi, Nepal, and Tanzania

Haiti								
	Mean difference <50	Std. Deviation	p-value	Mean difference <50 / Std. Deviation	Mean difference >50	Std. Deviation	p-value	Mean difference >50 / Std. Deviation
core simple v. core weighted	0.281	0.089	0.002	3.165	0.011	0.026	0.674	0.421
core simple v. core PCA	0.249	0.107	0.021	2.315	0.053	0.028	0.053	1.934
core simple v. expert simple	0.199	0.221	0.369	0.899	-0.027	0.026	0.296	-1.045
core simple v. expert weighted	0.196	0.087	0.024	2.255	0.001	0.028	0.984	0.019
core simple v. expert PCA	0.298	0.116	0.010	2.581	0.009	0.026	0.726	0.351
core simple v. maximum simple	-0.015	0.083	0.854	-0.184	0.012	0.028	0.664	0.434
core simple v. maximum weighted	0.100	0.078	0.200	1.283	0.062	0.029	0.032	2.150
core simple v. maximum PCA	0.077	0.111	0.487	0.695	0.039	0.026	0.134	1.497
core weighted v. core PCA	-0.032	0.095	0.735	-0.339	0.042	0.037	0.257	1.134
core weighted v. expert simple	-0.082	0.243	0.736	-0.337	-0.038	0.029	0.197	-1.291
core weighted v. expert weighted	-0.085	0.076	0.266	-1.113	-0.010	0.026	0.697	-0.390
core weighted v. expert PCA	0.018	0.110	0.873	0.159	-0.002	0.033	0.962	-0.048
core weighted v. maximum simple	-0.296	0.109	0.007	-2.714	0.001	0.034	0.971	0.037
core weighted v. maximum weighted	-0.181	0.079	0.021	-2.301	0.051	0.033	0.120	1.557
core weighted v. maximum PCA	-0.203	0.129	0.115	-1.575	0.028	0.036	0.440	0.773
core PCA v. expert simple	-0.050	0.261	0.849	-0.191	-0.080	0.034	0.019	-2.338
core PCA v. expert weighted	-0.053	0.101	0.602	-0.521	-0.053	0.035	0.131	-1.512
core PCA v. expert PCA	0.050	0.066	0.451	0.753	-0.044	0.023	0.058	-1.894
core PCA v. maximum simple	-0.264	0.120	0.028	-2.192	-0.041	0.040	0.304	-1.029
core PCA v. maximum weighted	-0.149	0.069	0.031	-2.155	0.008	0.038	0.827	0.219
core PCA v. maximum PCA	-0.171	0.101	0.091	-1.688	-0.015	0.029	0.614	-0.504
expert simple v. expert weighted	-0.003	0.196	0.988	-0.015	0.028	0.017	0.107	1.611
expert simple v. expert PCA	0.100	0.230	0.664	0.434	0.036	0.022	0.093	1.679
expert simple v. maximum simple	-0.214	0.225	0.343	-0.949	0.039	0.031	0.210	1.252
expert simple v. maximum weighted	-0.099	0.248	0.689	-0.400	0.089	0.034	0.008	2.635
expert simple v. maximum PCA	-0.121	0.231	0.600	-0.525	0.066	0.025	0.010	2.590
expert weighted v. expert PCA	0.103	0.088	0.242	1.169	0.009	0.024	0.712	0.369
expert weighted v. maximum simple	-0.211	0.098	0.032	-2.151	0.012	0.033	0.724	0.354
expert weighted v. maximum weighted	-0.096	0.079	0.223	-1.218	0.061	0.031	0.046	1.996
expert weighted v. maximum PCA	-0.118	0.109	0.280	-1.081	0.038	0.028	0.175	1.356
expert PCA v. maximum simple	-0.314	0.113	0.005	-2.777	0.003	0.035	0.935	0.082

expert PCA v. maximum weighted	-0.199	0.078	0.010	-2.560	0.052	0.035	0.138	1.483
expert PCA v. maximum PCA	-0.221	0.074	0.003	-2.988	0.029	0.022	0.181	1.338
maximum simple v. maximum weighted	0.115	0.079	0.145	1.457	0.049	0.023	0.031	2.157
maximum simple v. maximum PCA	0.093	0.085	0.276	1.089	0.026	0.025	0.285	1.069
maximum weighted v. maximum PCA	-0.022	0.084	0.792	-0.264	-0.023	0.029	0.419	-0.809
**Malawi**								
	Mean difference <50	Std. Deviation	p-value	Mean difference <50 / Std. Deviation	Mean difference >50	Std. Deviation	p-value	Mean difference >50 / Std. Deviation
core simple v. core weighted	0.014	0.193	0.943	0.071	0.118	0.045	0.009	2.619
core simple v. core PCA	-0.069	0.180	0.700	-0.386	0.102	0.057	0.073	1.792
core simple v. expert simple	-0.183	1.013	0.857	-0.181	-0.055	0.223	0.804	-0.249
core simple v. expert weighted	-0.101	0.299	0.736	-0.337	-0.002	0.046	0.966	-0.043
core simple v. expert PCA	-0.770	0.421	0.067	-1.829	-0.001	0.050	0.983	-0.021
core simple v. maximum simple	-0.632	0.359	0.079	-1.759	0.021	0.043	0.629	0.484
core simple v. maximum weighted	-0.558	0.203	0.006	-2.748	0.021	0.040	0.596	0.531
core simple v. maximum PCA	-0.462	0.190	0.015	-2.432	0.035	0.041	0.386	0.866
core weighted v. core PCA	-0.083	0.169	0.623	-0.492	-0.016	0.065	0.805	-0.247
core weighted v. expert simple	-0.197	0.995	0.843	-0.198	-0.174	0.227	0.443	-0.767
core weighted v. expert weighted	-0.115	0.294	0.697	-0.390	-0.120	0.043	0.005	-2.805
core weighted v. expert PCA	-0.784	0.417	0.060	-1.882	-0.119	0.060	0.047	-1.986
core weighted v. maximum simple	-0.645	0.416	0.120	-1.553	-0.097	0.055	0.078	-1.762
core weighted v. maximum weighted	-0.572	0.225	0.011	-2.538	-0.097	0.057	0.087	-1.711
core weighted v. maximum PCA	-0.476	0.222	0.032	-2.142	-0.083	0.059	0.159	-1.407
core PCA v. expert simple	-0.113	1.039	0.913	-0.109	-0.158	0.227	0.488	-0.694
core PCA v. expert weighted	-0.031	0.322	0.922	-0.098	-0.104	0.054	0.052	-1.942
core PCA v. expert PCA	-0.701	0.440	0.112	-1.591	-0.103	0.043	0.015	-2.421
core PCA v. maximum simple	-0.562	0.394	0.154	-1.425	-0.081	0.068	0.230	-1.200
core PCA v. maximum weighted	-0.489	0.210	0.020	-2.328	-0.081	0.064	0.206	-1.266
core PCA v. maximum PCA	-0.393	0.177	0.027	-2.214	-0.067	0.046	0.150	-1.438
expert simple v. expert weighted	0.082	0.901	0.927	0.091	0.054	0.221	0.808	0.243
expert simple v. expert PCA	-0.587	0.861	0.495	-0.682	0.054	0.220	0.805	0.247
expert simple v. maximum simple	-0.449	1.080	0.678	-0.415	0.076	0.223	0.731	0.344
expert simple v. maximum weighted	-0.376	1.044	0.719	-0.360	0.077	0.223	0.730	0.345
expert simple v. maximum PCA	-0.279	1.028	0.786	-0.271	0.091	0.223	0.683	0.408
expert weighted v. expert PCA	-0.669	0.345	0.053	-1.937	0.001	0.038	0.981	0.024

expert weighted v. maximum simple	-0.531	0.493	0.282	-1.076	0.023	0.053	0.665	0.433
expert weighted v. maximum weighted	-0.458	0.297	0.123	-1.543	0.023	0.048	0.626	0.487
expert weighted v. maximum PCA	-0.361	0.335	0.281	-1.079	0.037	0.049	0.448	0.759
expert PCA v. maximum simple	0.138	0.552	0.802	0.251	0.022	0.056	0.692	0.396
expert PCA v. maximum weighted	0.212	0.424	0.618	0.499	0.022	0.051	0.661	0.438
expert PCA v. maximum PCA	0.308	0.409	0.452	0.752	0.037	0.041	0.369	0.897
maximum simple v. maximum weighted	0.073	0.397	0.854	0.184	0.000	0.039	0.994	0.008
maximum simple v. maximum PCA	0.170	0.275	0.538	0.616	0.014	0.038	0.700	0.385
maximum weighted v. maximum PCA	0.096	0.216	0.655	0.448	0.014	0.039	0.717	0.362
**Nepal**								
	Mean difference <50	Std. Deviation	p-value	Mean difference <50 / Std. Deviation	Mean difference >50	Std. Deviation	p-value	Mean difference >50 / Std. Deviation
core simple v. core weighted	0.189	0.114	0.098	1.656	0.005	0.027	0.855	0.182
core simple v. core PCA	0.406	0.125	0.001*	3.261	-0.005	0.042	0.910	-0.113
core simple v. expert simple	-0.157	0.357	0.660	-0.439	-0.049	0.034	0.148	-1.446
core simple v. expert weighted	0.014	0.194	0.944	0.070	0.015	0.037	0.695	0.392
core simple v. expert PCA	0.220	0.214	0.304	1.028	-0.010	0.037	0.785	-0.272
core simple v. maximum simple	0.089	0.126	0.478	0.709	-0.064	0.034	0.061	-1.873
core simple v. maximum weighted	0.076	0.128	0.551	0.596	-0.007	0.031	0.825	-0.221
core simple v. maximum PCA	0.295	0.129	0.023	2.278	-0.090	0.041	0.028	-2.196
core weighted v. core PCA	0.217	0.130	0.096	1.664	-0.010	0.054	0.858	-0.179
core weighted v. expert simple	-0.346	0.380	0.362	-0.911	-0.054	0.040	0.182	-1.335
core weighted v. expert weighted	-0.175	0.185	0.342	-0.950	0.010	0.039	0.807	0.244
core weighted v. expert PCA	0.031	0.216	0.884	0.145	-0.015	0.045	0.736	-0.338
core weighted v. maximum simple	-0.100	0.147	0.497	-0.679	-0.069	0.048	0.152	-1.433
core weighted v. maximum weighted	-0.113	0.115	0.326	-0.982	-0.012	0.041	0.774	-0.288
core weighted v. maximum PCA	0.106	0.145	0.465	0.730	-0.094	0.055	0.088	-1.709
core PCA v. expert simple	-0.563	0.424	0.184	-1.328	-0.044	0.050	0.375	-0.887
core PCA v. expert weighted	-0.392	0.231	0.090	-1.697	0.019	0.060	0.748	0.322
core PCA v. expert PCA	-0.186	0.255	0.467	-0.728	-0.005	0.041	0.896	-0.131
core PCA v. maximum simple	-0.317	0.140	0.024	-2.262	-0.059	0.054	0.277	-1.087
core PCA v. maximum weighted	-0.330	0.115	0.004	-2.867	-0.002	0.057	0.972	-0.035
core PCA v. maximum PCA	-0.111	0.107	0.300	-1.036	-0.085	0.039	0.028	-2.196
expert simple v. expert weighted	0.171	0.269	0.526	0.634	0.064	0.027	0.017	2.383
expert simple v. expert PCA	0.377	0.295	0.200	1.281	0.039	0.024	0.099	1.648

expert simple v. maximum simple	0.246	0.369	0.504	0.668	-0.015	0.048	0.759	-0.307
expert simple v. maximum weighted	0.233	0.387	0.547	0.603	0.042	0.048	0.380	0.877
expert simple v. maximum PCA	0.452	0.374	0.227	1.207	-0.041	0.046	0.377	-0.883
expert weighted v. expert PCA	0.207	0.174	0.236	1.185	-0.025	0.037	0.509	-0.661
expert weighted v. maximum simple	0.076	0.199	0.703	0.381	-0.078	0.055	0.152	-1.433
expert weighted v. maximum weighted	0.063	0.190	0.742	0.330	-0.021	0.049	0.663	-0.436
expert weighted v. maximum PCA	0.281	0.190	0.139	1.480	-0.104	0.057	0.070	-1.813
expert PCA v. maximum simple	-0.131	0.248	0.597	-0.529	-0.053	0.053	0.318	-0.998
expert PCA v. maximum weighted	-0.144	0.233	0.536	-0.618	0.003	0.055	0.950	0.062
expert PCA v. maximum PCA	0.074	0.206	0.719	0.360	-0.079	0.042	0.057	-1.901
maximum simple v. maximum weighted	-0.013	0.087	0.881	-0.150	0.057	0.029	0.054	1.929
maximum simple v. maximum PCA	0.205	0.103	0.045	2.003	-0.026	0.039	0.504	-0.668
maximum weighted v. maximum PCA	0.218	0.108	0.043	2.026	-0.083	0.051	0.107	-1.610
**Tanzania**								
	Mean difference <50	Std. Deviation	p-value	Mean difference <50 / Std. Deviation	Mean difference >50	Std. Deviation	p-value	Mean difference >50 / Std. Deviation
core simple v. core weighted	0.339	0.121	0.005	2.812	0.055	0.019	0.004	2.848
core simple v. core PCA	0.099	0.075	0.189	1.315	0.061	0.011	0.000*	5.304
core simple v. expert simple	-0.324	0.413	0.433	-0.784	0.027	0.021	0.202	1.277
core simple v. expert weighted	-0.235	0.184	0.200	-1.281	0.042	0.023	0.068	1.822
core simple v. expert PCA	-0.072	0.128	0.573	-0.564	0.103	0.021	0.000*	4.809
core simple v. maximum simple	0.062	0.117	0.595	0.532	0.064	0.025	0.009	2.614
core simple v. maximum weighted	-0.018	0.116	0.875	-0.157	0.066	0.022	0.003	3.006
core simple v. maximum PCA	0.012	0.127	0.924	0.095	0.074	0.024	0.002	3.093
core weighted v. core PCA	-0.240	0.105	0.023	-2.281	0.006	0.023	0.805	0.247
core weighted v. expert simple	-0.663	0.423	0.117	-1.566	-0.028	0.027	0.294	-1.050
core weighted v. expert weighted	-0.575	0.183	0.002	-3.138	-0.013	0.025	0.602	-0.521
core weighted v. expert PCA	-0.411	0.141	0.004	-2.911	0.048	0.028	0.086	1.718
core weighted v. maximum simple	-0.277	0.132	0.035	-2.107	0.009	0.029	0.746	0.323
core weighted v. maximum weighted	-0.358	0.115	0.002	-3.107	0.011	0.025	0.658	0.443
core weighted v. maximum PCA	-0.327	0.130	0.012	-2.520	0.019	0.029	0.521	0.642
core PCA v. expert simple	-0.423	0.412	0.304	-1.027	-0.034	0.021	0.109	-1.601
core PCA v. expert weighted	-0.334	0.171	0.051	-1.956	-0.019	0.023	0.402	-0.837
core PCA v. expert PCA	-0.171	0.109	0.116	-1.571	0.043	0.017	0.012	2.512
core PCA v. maximum simple	-0.037	0.100	0.712	-0.369	0.004	0.025	0.886	0.143

core PCA v. maximum weighted	-0.117	0.094	0.209	-1.256	0.005	0.022	0.810	0.240
core PCA v. maximum PCA	-0.087	0.095	0.357	-0.920	0.013	0.021	0.539	0.615
expert simple v. expert weighted	0.089	0.408	0.828	0.217	0.015	0.019	0.441	0.770
expert simple v. expert PCA	0.252	0.356	0.479	0.707	0.076	0.013	0.000*	5.943
expert simple v. maximum simple	0.386	0.425	0.363	0.909	0.037	0.028	0.186	1.322
expert simple v. maximum weighted	0.305	0.429	0.476	0.713	0.039	0.028	0.170	1.373
expert simple v. maximum PCA	0.336	0.417	0.421	0.805	0.047	0.026	0.068	1.824
expert weighted v. expert PCA	0.163	0.164	0.319	0.996	0.061	0.021	0.003	2.984
expert weighted v. maximum simple	0.297	0.190	0.118	1.565	0.022	0.030	0.455	0.748
expert weighted v. maximum weighted	0.217	0.176	0.217	1.234	0.024	0.027	0.371	0.895
expert weighted v. maximum PCA	0.247	0.186	0.183	1.332	0.032	0.028	0.249	1.153
expert PCA v. maximum simple	0.134	0.123	0.274	1.093	-0.039	0.028	0.166	-1.385
expert PCA v. maximum weighted	0.054	0.131	0.683	0.409	-0.037	0.028	0.177	-1.351
expert PCA v. maximum PCA	0.084	0.121	0.487	0.694	-0.029	0.024	0.224	-1.215
maximum simple v. maximum weighted	-0.081	0.081	0.322	-0.989	0.002	0.017	0.919	0.102
maximum simple v. maximum PCA	-0.050	0.071	0.478	-0.710	0.010	0.016	0.535	0.620
maximum weighted v. maximum PCA	0.030	0.097	0.755	0.313	0.008	0.019	0.683	0.408

* p<=0.0014 (Bonferroni correction for multiple comparisons)

Supplementary Table 8: Client vs. Facility Comparison of Index Estimates, Haiti, Malawi, Nepal, Senegal, and Tanzania

	Mean difference	Std. Deviation	p-value	Mean difference/ Std. deviation
**Haiti**				
Core simple	0.034	0.026	0.200	1.281
Core weighted	0.023	0.022	0.291	1.057
Core PCA	0.033	0.019	0.074	1.787
Expert simple	0.034	0.024	0.166	1.385
Expert weighted	0.036	0.022	0.107	1.614
Expert PCA	0.033	0.019	0.083	1.735
Maximum simple	0.031	0.029	0.284	1.071
Maximum weighted	0.034	0.026	0.190	1.311
Maximum PCA	0.041	0.026	0.116	1.571
**Malawi**				
	Mean difference	Std. Deviation	p-value	Mean difference/ Std. deviation
Core simple	0.000	0.034	0.991	0.011
Core weighted	-0.007	0.027	0.802	-0.250
Core PCA	0.007	0.029	0.817	0.232
Expert simple	0.000	0.033	0.997	-0.004
Expert weighted	-0.004	0.031	0.892	-0.135
Expert PCA	0.008	0.035	0.831	0.213
Maximum simple	0.012	0.042	0.771	0.292
Maximum weighted	0.012	0.040	0.764	0.300
Maximum PCA	0.014	0.041	0.738	0.335
**Nepal**				
	Mean difference	Std. Deviation	p-value	Mean difference/ Std. deviation
Core simple	-0.004	0.033	0.899	-0.128
Core weighted	-0.013	0.030	0.672	-0.424
Core PCA	0.002	0.027	0.950	0.063
Expert simple	-0.005	0.034	0.870	-0.163
Expert weighted	-0.012	0.035	0.738	-0.334
Expert PCA	-0.003	0.030	0.929	-0.089
Maximum simple	0.018	0.039	0.648	0.457
Maximum weighted	-0.001	0.036	0.972	-0.035
Maximum PCA	0.023	0.035	0.519	0.645
**Senegal**				
	Mean difference	Std. Deviation	p-value	Mean difference/ Std. deviation
Core simple	0.011	0.037	0.772	0.290
Core weighted	0.008	0.026	0.753	0.314
Core PCA	0.020	0.058	0.733	0.341
Expert simple	0.009	0.042	0.833	0.210
Expert weighted	0.001	0.028	0.965	0.044

Expert PCA	0.016	0.097	0.872	0.162
Maximum simple	0.020	0.043	0.643	0.464
Maximum weighted	0.009	0.037	0.809	0.242
Maximum PCA	0.044	0.107	0.681	0.411
**Tanzania**				
	Mean difference	Std. Deviation	p-value	Mean difference/ Std. deviation
Core simple	-0.009	0.022	0.681	-0.411
Core weighted	-0.008	0.019	0.669	-0.427
Core PCA	-0.008	0.019	0.675	-0.419
Expert simple	-0.003	0.021	0.898	-0.128
Expert weighted	-0.013	0.022	0.550	-0.598
Expert PCA	-0.005	0.017	0.770	-0.293
Maximum simple	0.000	0.026	0.987	-0.016
Maximum weighted	-0.009	0.025	0.709	-0.373
Maximum PCA	-0.001	0.025	0.957	-0.054

* p<=0.006 (Bonferroni correction for multiple comparisons)

Supplementary Table 9: Facility Level Linear Model of the Association between Readiness and Provision of Care with a Spline at 50, Haiti, Malawi, Nepal, and Tanzania

					**Haiti**					
	N Ready <=50	Estimate <50	Std. Error	p-value	Estimate /Std. Error	N Ready >50	Estimate >50	Std. Error	p-value	Estimate /Std. Error
Core simple	78	0.347	0.128	0.007*	2.714	280	0.036	0.050	0.473	0.718
Core weighted	72	0.102	0.097	0.294	1.051	286	0.032	0.046	0.493	0.687
Core PCA	154	0.151	0.077	0.051	1.956	204	-0.006	0.044	0.883	-0.147
Expert simple	39	0.150	0.199	0.450	0.757	319	0.069	0.042	0.105	1.626
Expert weighted	72	0.196	0.112	0.082	1.743	286	0.039	0.044	0.374	0.890
Expert PCA	100	0.100	0.096	0.297	1.044	258	0.038	0.039	0.327	0.981
Maximum simple	82	0.330	0.139	0.019*	2.364	276	0.059	0.059	0.311	1.014
Maximum weighted	116	0.239	0.093	0.011*	2.572	242	0.011	0.060	0.860	0.177
Maximum PCA	82	0.245	0.142	0.086	1.723	276	0.022	0.051	0.672	0.424
					**Malawi**					
	N Ready <=50	Estimate <50	Std. Error	p-value	Estimate /Std. Error	N Ready >50	Estimate >50	Std. Error	p-value	Estimate /Std. Error
Core simple	54	0.206	0.242	0.395	0.851	199	0.163	0.075	0.031*	2.173
Core weighted	32	0.182	0.204	0.372	0.894	221	0.052	0.066	0.427	0.795
Core PCA	166	0.225	0.092	0.016*	2.431	87	0.067	0.077	0.382	0.876
Expert simple	6	0.502	0.772	0.516	0.651	247	0.206	0.065	0.002*	3.166
Expert weighted	18	0.392	0.362	0.280	1.084	235	0.167	0.062	0.007*	2.697
Expert PCA	21	1.051	0.484	0.031*	2.170	232	0.164	0.066	0.014*	2.479
Maximum simple	30	0.907	0.410	0.028*	2.210	223	0.124	0.074	0.092	1.690
Maximum weighted	35	0.762	0.304	0.013*	2.507	218	0.146	0.081	0.072	1.804
Maximum PCA	53	0.671	0.258	0.010*	2.603	200	0.119	0.076	0.117	1.572
					**Nepal**					
	N Ready <=50	Estimate <50	Std. Error	p-value	Estimate /Std. Error	N Ready >50	Estimate >50	Std. Error	p-value	Estimate /Std. Error
Core simple	56	0.203	0.013	2.487	0.203	226	0.021	0.067	0.759	0.307
Core weighted	42	0.153	0.058	1.903	0.153	240	0.030	0.066	0.648	0.457
Core PCA	78	0.123	0.408	0.828	0.123	204	0.015	0.071	0.829	0.216
Expert simple	10	0.460	0.167	1.387	0.460	272	0.071	0.063	0.265	1.116
Expert weighted	26	0.262	0.068	1.832	0.262	256	0.014	0.067	0.837	0.206
Expert PCA	25	0.268	0.368	0.902	0.268	257	0.032	0.056	0.565	0.577
Maximum simple	63	0.219	0.065	1.855	0.219	219	0.064	0.085	0.449	0.757

Maximum weighted	77	0.172	0.015	2.442	0.172	205	0.026	0.083	0.750	0.319
Maximum PCA	63	0.184	0.276	1.092	0.184	219	0.086	0.079	0.277	1.089
**Tanzania**										
	N Ready <=50	Estimate <50	Std. Error	p-value	Estimate /Std. Error	N Ready >50	Estimate >50	Std. Error	p-value	Estimate /Std. Error
Core simple	46	0.664	0.191	0.001*	3.475	586	0.169	0.040	0.000*	4.187
Core weighted	56	0.322	0.151	0.034*	2.128	576	0.112	0.036	0.002*	3.130
Core PCA	79	0.581	0.126	0.000*	4.628	553	0.109	0.039	0.005*	2.818
Expert simple	19	0.861	0.341	0.012*	2.527	613	0.142	0.037	0.000*	3.779
Expert weighted	32	0.934	0.246	0.000*	3.803	600	0.131	0.040	0.001*	3.275
Expert PCA	51	0.749	0.176	0.000*	4.247	581	0.065	0.033	0.051	1.952
Maximum simple	104	0.625	0.157	0.000*	3.971	528	0.094	0.051	0.065	1.847
Maximum weighted	104	0.699	0.145	0.000*	4.820	528	0.101	0.050	0.044*	2.022
Maximum PCA	96	0.675	0.159	0.000*	4.258	536	0.090	0.048	0.062	1.868

Notes: Model is a linear model with a spline at 50, controlling for facility characteristics; facility-level analysis
* p <0.05

Supplementary Table 10: Client v. Facility Comparison of Index Estimates with Spline, Haiti, Malawi, Nepal, and Tanzania

	Mean difference <50	Std. Deviation	p-value	Mean difference <50 / Std. Deviation	Mean difference >50	Std. Deviation	p-value	Mean difference >50 / Std. Deviation
**Haiti**								
Core simple	0.042	0.087	0.628	0.484	0.036	0.032	0.258	1.131
Core weighted	0.022	0.062	0.723	0.355	0.024	0.030	0.421	0.804
Core PCA	0.068	0.047	0.150	1.438	0.020	0.029	0.489	0.692
Expert simple	0.046	0.128	0.722	0.355	0.032	0.027	0.227	1.207
Expert weighted	0.034	0.071	0.628	0.485	0.038	0.027	0.167	1.381
Expert PCA	0.061	0.059	0.299	1.038	0.026	0.024	0.271	1.100
Maximum simple	0.089	0.092	0.334	0.967	0.019	0.038	0.615	0.503
Maximum weighted	0.076	0.056	0.174	1.361	0.019	0.039	0.633	0.478
Maximum PCA	0.110	0.084	0.190	1.310	0.029	0.033	0.378	0.881
**Malawi**								
	Mean difference <50	Std. Deviation	p-value	Mean difference <50 / Std. Deviation	Mean difference >50	Std. Deviation	p-value	Mean difference >50 / Std. Deviation
Core simple	-0.041	0.129	0.752	-0.316	0.008	0.045	0.863	0.173
Core weighted	-0.037	0.117	0.749	-0.320	-0.001	0.039	0.980	-0.026
Core PCA	-0.001	0.054	0.982	-0.022	0.013	0.040	0.740	0.331
Expert simple	0.022	0.412	0.957	0.055	0.001	0.096	0.995	0.007
Expert weighted	-0.030	0.215	0.890	-0.138	-0.001	0.035	0.967	-0.041
Expert PCA	0.023	0.259	0.930	0.087	0.008	0.037	0.820	0.228
Maximum simple	-0.160	0.273	0.558	-0.586	0.026	0.046	0.571	0.566
Maximum weighted	-0.001	0.157	0.993	-0.009	0.016	0.049	0.744	0.327
Maximum PCA	-0.044	0.149	0.765	-0.299	0.024	0.045	0.596	0.530
**Nepal**								
	Mean difference <50	Std. Deviation	p-value	Mean difference <50 / Std. Deviation	Mean difference >50	Std. Deviation	p-value	Mean difference >50 / Std. Deviation
Core simple	-0.025	0.116	0.832	-0.213	0.003	0.041	0.951	0.062
Core weighted	-0.005	0.095	0.962	-0.048	-0.014	0.040	0.725	-0.351
Core PCA	-0.051	0.076	0.508	-0.662	0.028	0.044	0.516	0.649
Expert simple	-0.005	0.199	0.980	-0.026	-0.005	0.036	0.890	-0.138
Expert weighted	0.021	0.128	0.868	0.166	-0.015	0.039	0.704	-0.380
Expert PCA	-0.009	0.167	0.956	-0.055	-0.002	0.031	0.960	-0.050
Maximum simple	-0.055	0.113	0.627	-0.486	0.038	0.051	0.458	0.743
Maximum weighted	-0.035	0.085	0.681	-0.411	0.012	0.050	0.808	0.244
Maximum PCA	-0.059	0.104	0.568	-0.572	0.047	0.046	0.299	1.040

Tanzania								
	Mean difference <50	Std. Deviation	p-value	Mean difference <50 / Std. Deviation	Mean difference >50	Std. Deviation	p-value	Mean difference >50 / Std. Deviation
Core simple	0.037	0.088	0.674	0.420	-0.013	0.025	0.614	-0.504
Core weighted	0.059	0.084	0.481	0.704	-0.018	0.023	0.435	-0.781
Core PCA	0.013	0.064	0.839	0.203	-0.010	0.023	0.676	-0.417
Expert simple	0.009	0.211	0.968	0.041	-0.003	0.021	0.900	-0.126
Expert weighted	-0.042	0.138	0.758	-0.308	-0.011	0.024	0.634	-0.476
Expert PCA	-0.008	0.095	0.935	-0.082	-0.003	0.019	0.885	-0.144
Maximum simple	0.007	0.087	0.940	0.075	0.006	0.031	0.857	0.180
Maximum weighted	0.016	0.079	0.839	0.203	-0.009	0.031	0.772	-0.289
Maximum PCA	-0.001	0.092	0.989	-0.013	0.005	0.029	0.856	0.182

* p<=0.006 (Bonferroni correction for multiple comparisons)
